# Strictosidine activation in Apocynaceae: towards a "nuclear time bomb"?

**DOI:** 10.1186/1471-2229-10-182

**Published:** 2010-08-19

**Authors:** Grégory Guirimand, Vincent Courdavault, Arnaud Lanoue, Samira Mahroug, Anthony Guihur, Nathalie Blanc, Nathalie Giglioli-Guivarc'h, Benoit St-Pierre, Vincent Burlat

**Affiliations:** 1 Université François Rabelais de Tours, EA 2106 "Biomolécules et Biotechnologies Végétales"; IFR 135 "Imagerie fonctionnelle" 37200, Tours, France; 2 Laboratoire Biodiversité Végétale, Conservation et Valorisation, Faculté des Sciences, Université Djillali Liabés, Sidi Bel Abbes, Algérie; 3 Université de Toulouse; UPS; UMR 5546, Surfaces Cellulaires et Signalisation chez les Végétaux; BP 42617, F-31326, Castanet-Tolosan, France; 4 CNRS; UMR 5546; BP 42617, F-31326, Castanet-Tolosan, France

## Abstract

**Background:**

The first two enzymatic steps of monoterpene indole alkaloid (MIA) biosynthetic pathway are catalysed by strictosidine synthase (STR) that condensates tryptamine and secologanin to form strictosidine and by strictosidine β-D-glucosidase (SGD) that subsequently hydrolyses the glucose moiety of strictosidine. The resulting unstable aglycon is rapidly converted into a highly reactive dialdehyde, from which more than 2,000 MIAs are derived. Many studies were conducted to elucidate the biosynthesis and regulation of pharmacologically valuable MIAs such as vinblastine and vincristine in *Catharanthus roseus *or ajmaline in *Rauvolfia serpentina*. However, very few reports focused on the MIA physiological functions.

**Results:**

In this study we showed that a strictosidine pool existed *in planta *and that the strictosidine deglucosylation product(s) was (were) specifically responsible for *in vitro *protein cross-linking and precipitation suggesting a potential role for strictosidine activation in plant defence. The spatial feasibility of such an activation process was evaluated *in planta*. On the one hand, *in situ *hybridisation studies showed that CrSTR and CrSGD were coexpressed in the epidermal first barrier of *C. roseus *aerial organs. However, a combination of GFP-imaging, bimolecular fluorescence complementation and electromobility shift-zymogram experiments revealed that STR from both *C. roseus *and *R. serpentina *were localised to the vacuole whereas SGD from both species were shown to accumulate as highly stable supramolecular aggregates within the nucleus. Deletion and fusion studies allowed us to identify and to demonstrate the functionality of CrSTR and CrSGD targeting sequences.

**Conclusions:**

A spatial model was drawn to explain the role of the subcellular sequestration of STR and SGD to control the MIA metabolic flux under normal physiological conditions. The model also illustrates the possible mechanism of massive activation of the strictosidine vacuolar pool upon enzyme-substrate reunion occurring during potential herbivore feeding constituting a so-called "nuclear time bomb" in reference to the "mustard oil bomb" commonly used to describe the myrosinase-glucosinolate defence system in Brassicaceae.

## Background

**Figure 1:**
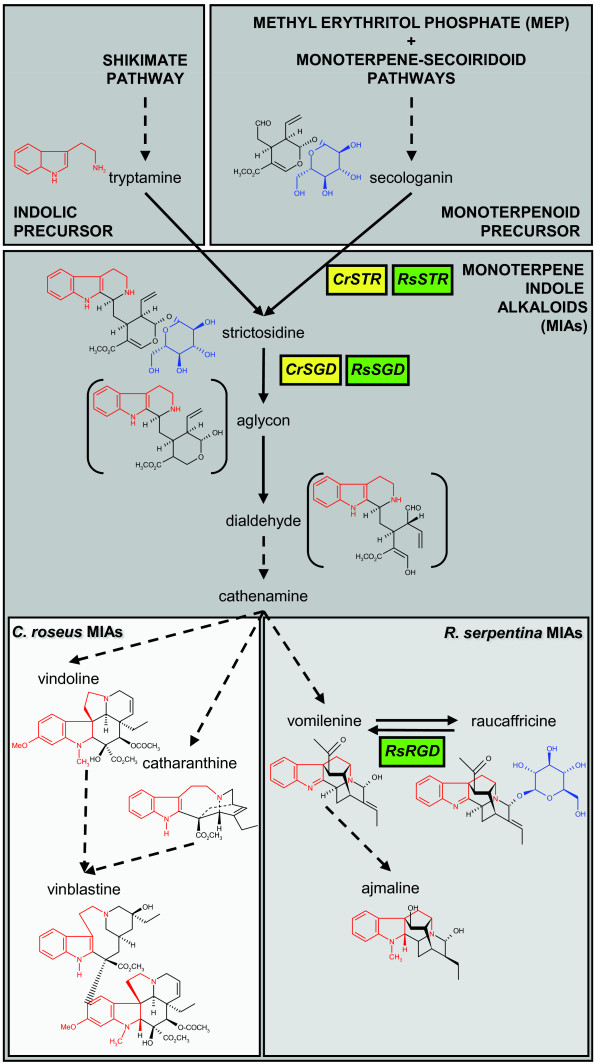
**Monoterpene indole alkaloid (MIA) biosynthetic pathway showing the common entry to the pathway through the biosynthesis of strictosidine and its subsequent deglucosylation leading to species-specific end-products identified in *Catharanthus roseus *and *Rauvolfia serpentina***. The biosynthetic pathways of MIA precursors and the first MIA biosynthetic steps are common to all MIA-producing species (dark grey background). The species-specific MIA chemical diversity is exemplified in *C. roseus *and *R. serpentina *(light greys background) where major advances in molecular biology of MIA biosynthesis have been performed. Chemical structures are coloured in red (indolic moieties), in black (monoterpenoid moieties) and in blue (glucose). Cr, *C. roseus *(yellow); Rs, *R. serpentina *(green); STR, strictosidine synthase; SGD, strictosidine β-D-glucosidase; RGD, raucaffricine β-D-glucosidase.

Strictosidine and its aglycon are the common first two monoterpene indole alkaloids (MIAs) giving rise to more than 2,000 specific MIAs in different plant species [1-5] (Figure 1). Some species-specific MIAs such as vinblastine and vincristine in *Catharanthus roseus *or ajmaline in *Rauvolfia serpentina *are well known for their highly valuable pharmaceutical properties [4-6] (Figure 1). Until now, few studies have addressed the physiological role of MIAs even though their high cytotoxicity points towards a role in plant defence [7-9].

The first committed step in MIA biosynthesis is carried out by strictosidine synthase (STR; EC: 4.3.3.2) which catalyses the condensation of the indolic precursor tryptamine with the glucosylated secoiridoid precursor secologanin to produce strictosidine (Figure 1). Subsequently, strictosidine β-D-glucosidase (SGD; EC: 3.2.1.105) hydrolyses the strictosidine glucose moiety producing an unstable aglycon that is rapidly converted into a dialdehyde intermediate and further into cathenamine [1,2,6,10] (Figure 1). Following an uncomplete *C. roseus *STR sequence description [11], the full cDNA encoding these enzymes have been isolated from both *C. roseus *[1,12] and *R. serpentina *[2,13].

Glycoside hydrolysis by specific sequestrated glycosidases activates many glycosylated secondary metabolites leading to plant defence strategies against herbivores [14] such as those observed in the so-called "mustard oil bomb" glucosinolate-myrosinase defence systems in Brassicaceae [15-18]. Although the differential compartmentation has not been elucidated in every model, the accumulating glucosylated metabolites must be physically separated (either at the cellular level or at the subcellular level) from the activating β-glucosidases [18]. The activation of toxic or repulsive metabolites occurs following enzyme-substrate reunion during herbivore feeding [14]. Such an activation mechanism has been proposed for strictosidine with the formulated hypothesis that upon cell damage, SGD would rapidly convert strictosidine into an aglycon [1,2,9], which has been shown to have antimicrobial activity [9]. However, no formal demonstration of such a process has been published so far. Interestingly, studies on *Ligustrum obtusifolium *leaves showed that an unidentified sequestrated β-glucosidase was able to activate a compound chemically related to strictosidine, *i.e*. the phenolic secoiridoid glucoside oleuropein, leading to the production of an highly reactive dialdehyde that acts as a strong protein cross-linker with a potent chemical defence role [19].

In this work, we evaluated the feasibility of such an enzymatic activation mechanism for strictosidine with the production of the dialdehyde intermediate by SGD. Our efforts were focused mainly on the *C. roseus *model and to a lesser extent on the *R. serpentina *enzymes. Electrophoretic-mobility shift assays (EMSA) clearly show that the strictosidine deglucosylation product(s) has/have *in vitro *protein cross-linking and precipitating properties that strictosidine does not have. We therefore carefully studied, using *in situ *hybridisation and GFP-imaging approaches, the cellular and subcellular localisation of STR and SGD to ascertain the physical separation of both enzymes. Our results reveal a common localisation of both gene products in the *C. roseus *epidermis, with STR being sequestrated in the vacuole whereas SGD intriguingly accumulated as highly stable supramolecular aggregates within the nucleus. The results are discussed both in terms of physiological and ecophysiological perspectives.

## Results and discussion

### The strictosidine deglucosylation product(s) promote(s) *in vitro *protein cross-linking and precipitation

**Figure 2:**
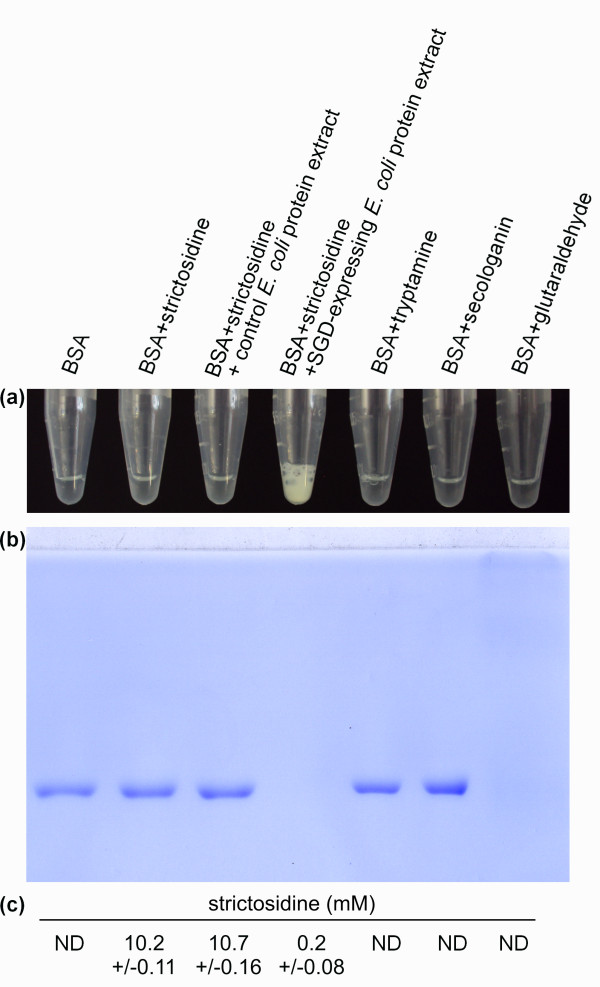
**The specific CrSGD-mediated deglucosylation activates strictosidine and induces *in vitro *cross-linking and precipitation of BSA**. (a) Picture of microtubes showing the apparition of a yellowish precipitate within few seconds when CrSGD was mixed with 10 mM strictosidine, its specific substrate. (b) EMSA (SDS-PAGE and Coomassie blue staining) of supernatants showing that in the specific presence of both strictosidine and CrSGD the BSA was cross-linked and precipitated and therefore barely entered the gel with a consecutive disappearance of the monomeric BSA band at 66 kDa. Cross-linking of BSA with glutaraldehyde is also shown as a control. (c) HPLC monitoring of the final strictosidine concentration (mM) in the supernatants allowed to check the specificity of strictosidine deglucosylation by recombinant CrSGD. ND, not determined.

The first step of this study consisted of testing the ability of CrSGD to promote protein cross-linking *in vitro *using EMSA with BSA as a standard protein (Figure 2). A typical yellowish precipitate [20] specifically appeared when 10 mM strictosidine and the CrSGD extract were mixed (Figure 2a). The EMSA supernatant analysis revealed that the BSA migrated correctly displaying a typical 66 kDa band with similar Coomassie blue staining intensities under the different control conditions, *i.e*. BSA alone or in presence of strictosidine, BSA in presence of strictosidine and a control *E. coli *protein extract, as well as BSA in presence of tryptamine or secologanin, the two strictosidine precursors (Figures 1 and 2b) and BSA in presence of a protein extract from CrSGD-overexpressing *E. coli *cells (data not shown). Interestingly, the 66 kDa BSA band disappeared when BSA was incubated with strictosidine and a protein extract from CrSGD-overexpressing *E. coli *cells in a similar way to BSA incubated with glutaraldehyde which is used as a protein cross-linking control (Figure 2b). Under these two conditions, most of the BSA was retained in the pellet since only traces of high molecular weight cross-linked BSA barely entered the gel. However, the heavy yellowish precipitation was specific to the SGD/strictosidine condition since it was not observed with glutaraldehyde. The HPLC monitoring of strictosidine disappearance confirmed the specificity of the CrSGD enzymatic reaction and excluded the possibility of non-specific degradation of strictosidine (Figure 2c). Similar results were also obtained even if less spectacular using 1 mM strictosidine (data not shown). From these *in-vitro *results it was hypothesised that the specific strictosidine deglucosylation leading to a dialdehyde aglycon could have evolved as an activation process leading to protein cross-linking and precipitation that may help *C. roseus *in defence processes against herbivore attacks in a similar way as during the activation of the phenolic secoiridoid glycoside oleuropein in *L*. *obtusifolium *[19].

### *CrSTR *and *CrSGD *are co-expressed in the epidermis of aerial organs

**Figure 3:**
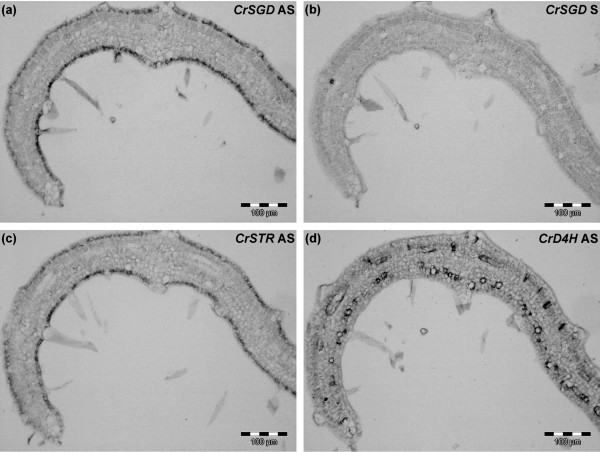
***CrSGD *is expressed in the epidermis of *C. roseus *aerial organs**. Serial longitudinal sections of a young developing leaf were hybridised either with *CrSGD *antisense (AS) riboprobes, with *CrSGD *sense (S) probes used as a negative control or with a *CrSTR *AS probe and a *CrD4H *AS probe used as positive controls [28]. In the revoluted developing base of the leaf, *CrSGD *is specifically coexpressed in the epidermis with *CrSTR*, the preceding gene in the monoterpene indole alkaloid (MIA) pathway whereas *CrD4H *involved in a downstream step along the MIA pathway is expressed in the specialised laticifer-idioblast cells. *Cr*, *C. roseus*; *STR*, strictosidine synthase; *SGD*, strictosidine β-D-glucosidase; *D4H*, desacetoxyvindoline 4-hydroxylase. Bar: 100 μm

The *in planta *feasibility of the strictosidine activation following potential herbivore feeding necessitated a spatial separation of strictosidine and CrSGD within distinct cell types and/or distinct organelles. In *C. roseus*, the MIA biosynthetic pathway displays a complex multicellular spatial organisation with at least three groups of cell types being involved. The early steps of the monoterpenoid pathway occur within the internal phloem associated parenchyma specialised cells [21-25]. Gene products from tryptophan decarboxylase (CrTDC), secologanin synthase (CrSLS), involved in the synthesis of tryptamine and secologanin, respectively, as well as from CrSTR are specifically localised to the epidermis [25-28]. Finally, the last two steps in vindoline biosynthesis take place within specialised laticifer and idioblast cells [28]. No direct evidence of CrSGD cell-specific localisation was available and it was therefore not possible to determine whether or not CrSTR and CrSGD were located in different cell types. Thus, the localisation of CrSTR and CrSGD genes products were first studied at the cellular level. Using *in situ *hybridisation, we clearly demonstrated that both genes were specifically expressed within the epidermis (Figure 3a-c) and not within the laticifer-idioblast CrD4H-expressing cells (Figure 3d). This result was in agreement with recent transcriptomic analysis showing a relative enrichment of CrSGD expression in the *C. roseus *epidermome [29]. This result also reinforced the definition of epidermis as a pivotal site for secondary metabolism in *C. roseus *[27] and suggested that the epidermis as first barrier was involved in strictosidine-mediated defence processes. Therefore, it was relevant to investigate the existence of a compartmentation between the site of strictosidine synthesis and the site of strictosidine deglucosylation among the different organelles of epidermal cells.

### CrSTR is targeted to the vacuole through the secretory pathway allowing vacuolar strictosidine accumulation

**Figure 4:**
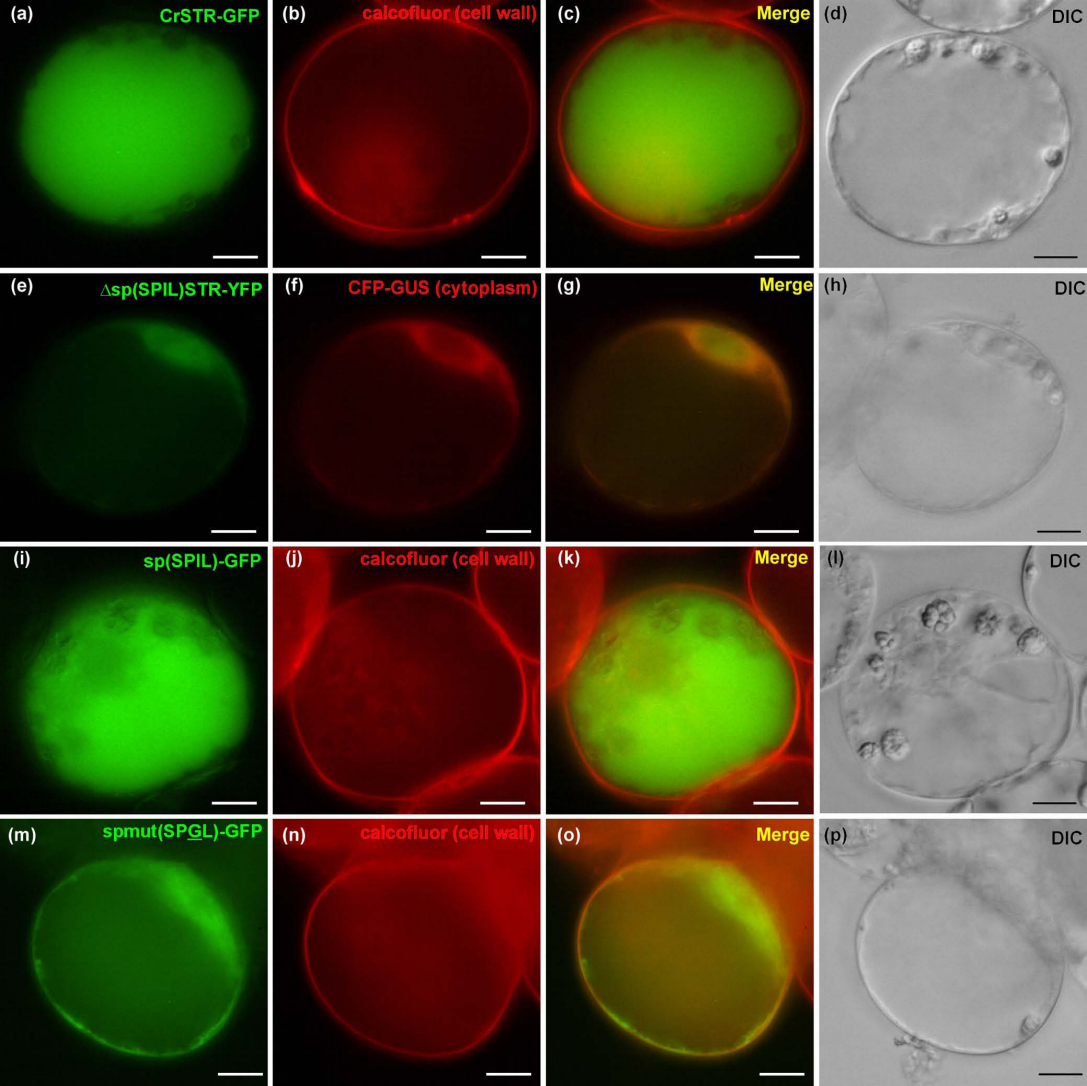
**CrSTR is targeted to the vacuole of *C. roseus *cells by an N-terminal signal peptide and a consecutive vacuolar sorting tetrapeptide (SPIL)**. Undifferentiated *C. roseus *cells were transformed to express CrSTR-GFP or deletion/fusion GFP constructs as indicated in the 1^st ^column. Calcofluor and CFP-GUS were used to visualise the cell wall and the cytoplasm, respectively (2^nd ^column). The merged image and the DIC morphology are presented in the 3^rd ^and 4^th ^columns, respectively. Note that CrSTR-GFP was localised to the vacuole and that the N-terminus signal peptide was necessary and sufficient to target the fusion protein to the secretory pathway whereas the tetrapetide SPIL was necessary and sufficient to further sort to the vacuole the fusion protein that entered the secretory pathway. sp(SPIL), N-terminus CrSTR signal peptide and SPIL vacuolar sorting tetrapeptide; spmut(SPGL), N-terminus CrSTR signal peptide and mutated SPGL vacuolar sorting tetrapeptide; Δsp(SPIL)STR, CrSTR deleted from sp(SPIL). Bar: 10 μm.

**Figure 5:**
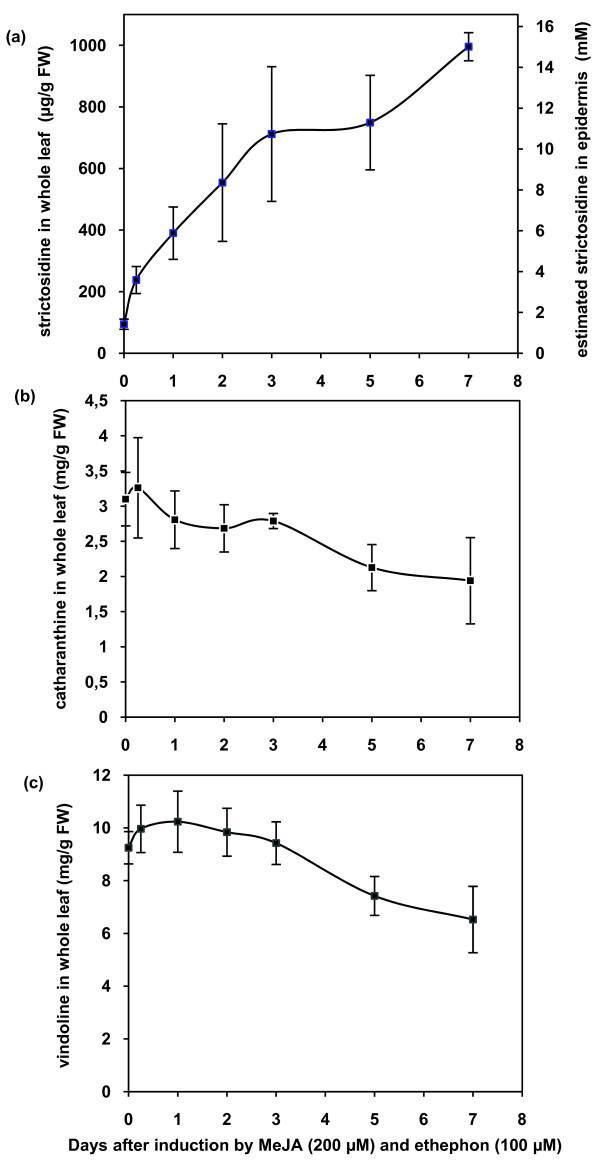
**Time course of strictosidine (a), catharanthine (b) and vindoline (c) contents in *C. roseus *leaves following MeJa and Ethephon treatment**. The results for strictosidine, catharanthine and vindoline are expressed in mg/g FW of whole leaves (left y axis) and for strictosidine a calculated estimation of concentration (mM) within the epidermis is provided (right y axis). This estimation takes into account the molecular weight of strictosidine (530.56 g.mol^-1^), a rough estimation that 80% of the fresh weight is liquid, and a 10-fold enrichment for the epidermis fraction as compared to the whole leaves deduced from [29].

Contradictory density gradient and immunogold analysis reported CrSTR as being either surprisingly cytoplasmic [30] or vacuolar [31,32]. Therefore, we used the recently optimised biolistic-mediated transient transformation of *C. roseus *cells applied to GFP-imaging [24] to illustrate the putative vacuolar CrSTR localisation but also to identify potential vacuolar targeting sequences. Indeed, CrSTR-GFP appeared to accumulate within the vacuole (Figure 4a-d) as previously suggested [31,32]. This was also in agreement with the predicted N-terminal signal peptide (sp) (1-MANFSESKSMMAVFFMFFLLLLSSSSSSSSS-31) followed by a vacuolar sorting-like sequence (32-SPIL-35) reminiscent of NPIR(L) vacuolar targeting N-terminal sequence of papain-like cysteine proteases from various plant species and sweet potato sporamin [33,34]. Accordingly, a CrSTR N-terminus truncated version deprived from the N-terminal signal peptide and the vacuolar sorting-like tetrapeptide was neither able to enter the secretory pathway or reach the vacuole and consequently remained in the cytoplasm with a passive diffusion to the nucleus (Figure 4e-h). In turn, the sole N terminal sequence was able to specifically drive the targeting of GFP to the vacuole (Figure 4i-l). Conversely, a single mutation (SPGL) within the putative vacuolar sorting sequence (SPIL) disabled the targeting of the fusion protein to the vacuole and rerouted it to the secretory pathway towards the plasma membrane-cell wall region (Figure 4m-p) similarly to what was described in the case of sporamin [34]. Finally, treatment with Brefeldin A (BFA), a drug disrupting the ER-to-cis golgi anterograde endomembrane transport system [35], led to the retention of CrSTR-GFP within the BFA-induced disorganised ER (Additional file 1). This illustrates that the CrSTR vacuolar targeting was carried out via the classical ER-to-golgi-to-vacuole route. The fact that strictosidine was synthesised in the vacuole does not necessarily imply that a strictosidine pool exists. Therefore, we studied the strictosidine, catharanthine and vindoline contents within whole leaves of 13 week-old *C. roseus in vitro *plants using HPLC (Figures 1 and 5). We measured 94 ± 16 μg of strictosidine per g of fresh young leaves in relative agreement with the detection of 63 ± 29 and 260 ± 30 μg per g of fresh mature and young leaves from greenhouse-raised mature *C. roseus*, respectively [9]. Moreover, under our conditions, the strictosidine leaf content increased following methyljasmonate (MeJa) and ethephon treatment mimicking herbivore and/or necrotrophic microorganism attack to reach 995 ± 45 μg.g^-1 ^of fresh weight within one week (Figure 5a). However, the catharanthine and vindoline contents were constitutively higher and slightly decreased following hormonal treatments (Figures 5b-c). In order to better compare these strictosidine concentrations with the condition used in our *in vitro *EMSA tests (Figure 2), we also estimated the results in mM strictosidine within the epidermis (Figure 5a) since both STR and SGD were localised to this cell type (Figure 3). The unit conversion takes into account the molecular weight of strictosidine (530.56 g.mol^-1^), a rough estimation that at least 80% of the leave fresh weight correspond to aqueous vacuoles, and a 10-fold enrichment factor for the epidermis fraction as compared to the whole leaves deduced from [29]. Indeed, concerning this last parameter, the recent *C. roseus *epidermome analysis revealed a 10-fold enrichment of the enzymatic activities of loganic acid *O*-methyltransferase (LAMT) involved in secologanin biosynthesis and 16-hydroxytabersonine 16-*O*-methyltransferase (16OMT) involved in vindoline biosynthesis, within the epidermome fraction of *C. roseus *young leaves as compared to the whole young *C. roseus *fraction [29]. As a consequence, it is reasonable to think that the strictosidine content within the epidermis was at least 1.4 mM under normal *in vitro *conditions and reached 15 mM following hormonal treatment mimicking herbivore attack (Figure 5a). Therefore these values constitute a significant strictosidine pool and somehow validate the physiological relevance of the *in vitro *concentration (10 mM and to a lesser extend 1 mM) used during the EMSA experiments (Figure 2).

### CrSGD is targeted to the nucleus using a bipartite NLS sequence and adopts a multimerised organisation relying on an accessible C-terminus sequence

**Figure 6:**
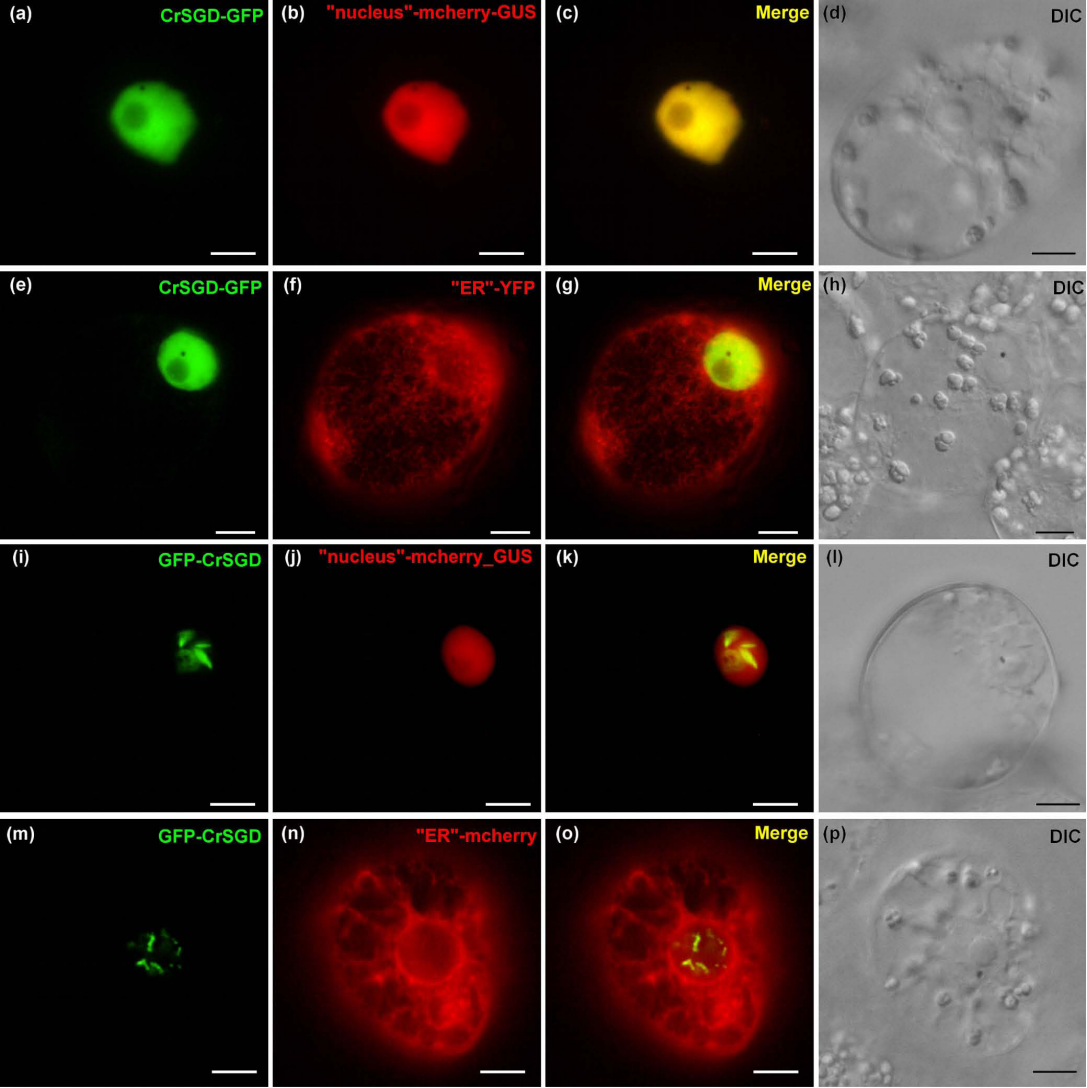
**CrSGD is localised to the nucleus of *C. roseus *cells with a diffuse or a punctuated/fusiform aggregation-like pattern of fluorescence dependant on the orientation of the GFP fusion**. Undifferentiated *C. roseus *cells were co-transformed with CrSGD-GFP or GFP-CrSGD constructs (1^st ^column) together with mcherry or YFP organelle markers constructs (2^nd ^column). The merged image and the DIC morphology are presented in the 3^rd ^and 4^th ^columns, respectively. Note that SGD fusions were localised to the nucleus and not to the ER with either a diffuse fluorescence pattern (CrSGD-GFP) and a punctuated or fusiform aggregation-like pattern of fluorescence (GFP-CrSGD). Bar: 10 μm.

**Figure 7:**
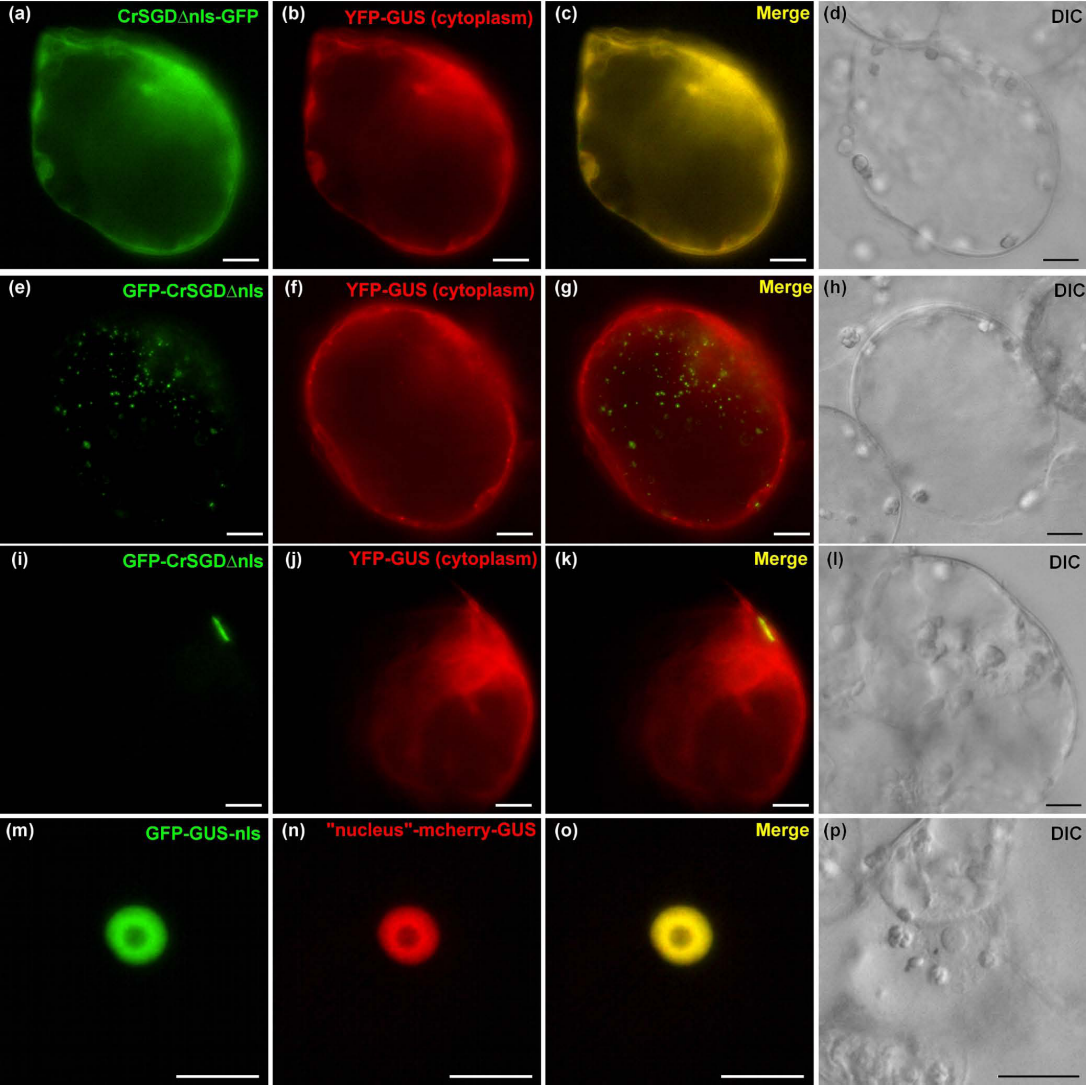
**The C-terminal CrSGD bipartite nuclear localisation signal (NLS) is necessary and sufficient to drive the nuclear targeting of CrSGD and an accessible NLS-independent CrSGD sequence located upstream to the NLS promotes the aggregation of CrSGD**. Undifferentiated *C. roseus *cells were co-transformed to express deletion/fusion forms of CrSGD (1^st ^column) and organelle markers (2^nd ^column). The merged image and the DIC morphology are presented in the 3^rd ^and 4^th ^columns, respectively. Note that the bipartite NLS from CrSGD was necessary for the nuclear targeting since fusion protein deprived from the NLS remained in the cytoplasm either displaying a diffuse pattern of fluorescence when the GFP was fused in the C-terminus extremity of the deleted CrSGD (a-d), or a punctuated (e-h) or fusiform (i-l) pattern of fluorescence when the deleted CrSGD C-terminus extremity was accessible in the fusion protein. Note also that the bipartite NLS was sufficient to drive the nuclear targeting but unable to promote the aggregation pattern (m-p). Bar: 10 μm.

**Figure 8:**
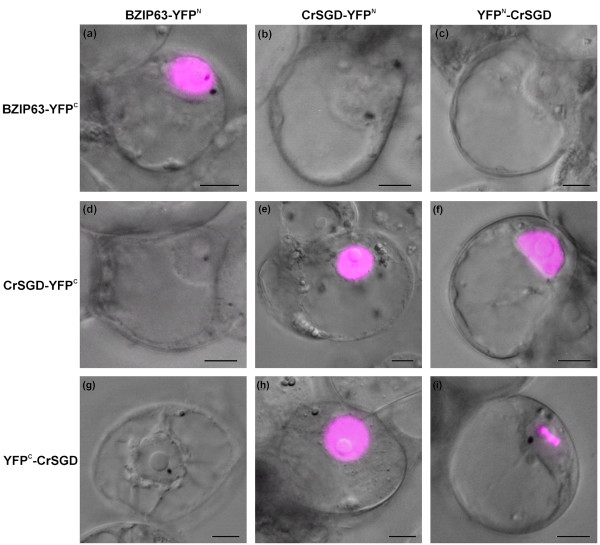
**BiFC experiments showing that CrSGD fused either in N-terminus or C-terminus of YFP fragments are able to specifically interact**. To evaluate the BiFC, undifferentiated *C. roseus *cells were co-transformed with the construct indicated on the left (fusions with the YFP^C ^fragment) and the construct indicated on the top (fusions with the YFP^N ^fragment). Interactions between two CrSGD fusions allowed reconstitution of the YFP fluorochrome with a diffuse nuclear fluorescence pattern when at least one of the C-terminus was blocked by a YFP fragment (e, f, h), whereas an aggregated fluorescence pattern required the interaction of two fusion proteins with an accessible CrSGD C-terminal extremity. bZIP63 was used as a positive control of BiFC and to test for non-specific interaction with CrSGD fusions. The efficiency of the co-transformation was further evaluated with the co-transformation of a "plastid"-CFP marker (Additional file 4). The images presented are merges of the YFP BiFC channel (Magenta false colour) and DIC channel to show the morphology of the cells. YFP^N^, (aa 1-173); YFP^C^, (aa 156-239). Bar: 10 μm.

CrSGD subcellular localisation appears to be unclear. It was indirectly hypothesised to be localised to the ER based on *in vivo *apparition of strictosidine-induced yellow fluorescence in a cell domain described as the ER in *C. roseus *cells and protoplasts and on the presence of a putative ER-anchoring KKXKX C-terminal sequence [1]. The same authors also noticed putative peroxisome internal targeting sequences. We used the GFP imaging approach to further study the CrSGD subcellular localisation. Surprisingly, in *C. roseus *cells, CrSGD-GFP perfectly colocalised with the nucleus marker (Figure 6a-d) and was clearly excluded from the ER (Figure 6e-h) contradicting the previous proposed CrSGD ER localisation [1]. Careful examination of the CrSGD 555 residues allowed us to identify a C-terminal bipartite Nuclear Localisation Signal (NLS) (537-K**KR**FREEDKLVELV**KK**Q**K**Y-555) that included the putative KKXKX C-terminal sequence [1]. This sequence matched exactly with the bipartite NLS consensus sequence (Prosite, PDOC00015) composed of two basic residues (K/R) followed by a spacer of up to 10 residues and ending with a 5 residue-sequence including at least three basic residues (K/R) as originally described for nucleoplasmin [36]. The reverse fusion construct, leaving the bipartite NLS accessible (GFP-CrSGD), was still targeted to the nucleus and not to the ER, but with a punctuated fluorescence pattern (data not shown) rapidly switching to a fusiform fluorescence pattern as the time of protein expression increased (Figure 6i-p). Such aggregation-like pattern was in agreement with the proposed CrSGD multimerised organisation [1,10,37]. In order to ascertain that this nuclear localisation actually corresponded to the fusion proteins and was not due to an improbable passive diffusion of GFP within the nucleus following cleavage from the fusion proteins, SDS-PAGE and anti-GFP western blots were performed on two *C. roseus *cell lines that were stably transformed with CrSGD-GFP and GFP-CrSGD constructs, respectively (Additional file 2a) and that constitutively displayed the same diffuse versus aggregated nuclear fluorescence patterns as the corresponding transiently transformed cells (Additional file 2b). Accordingly, a single band around 95 kDa was obtained with both CrSGD-GFP and GFP-CrSGD in close agreement with the theoretical 91 kDa of the fusions based on the apparent 63 kDa of SGD [1] and the 28 kDa of GFP (Additional file 2a). Additionally, the respective subcellular localisation of CrSGD-GFP and GFP-CrSGD were confirmed within *C. roseus *leaf epidermal cells, *i.e*. the *in planta CrSGD*-expressing cells (Additional file 3a-h). Therefore, a functional analysis of the bipartite NLS was conducted by either NLS deletion or fusion experiments. The bipartite NLS appeared necessary for the nuclear targeting but unnecessary for the aggregation like-pattern. Indeed, CrSGDΔnls-GFP was unable to reach the nucleus and perfectly colocalised with the cytoplasm marker YFP-GUS (Figure 7a-d) whereas GFP-CrSGDΔnls also remained in the cytoplasm with rather a punctuated (Figure 7e-h) and eventually a fusiform (Figure 7i-l) cytoplasmic fluorescence pattern. Furthermore, the bipartite NLS was also sufficient for nuclear targeting since GFP-GUS-nls displayed a nuclear localisation (Figure 7m-p). The interaction of CrSGD within the nucleus was further studied by Bimolecular Fluorescence Complementation (BiFC) in *C. roseus *cells (Figure 8; Additional file 4). Positive control of protein-protein specific interaction in the nucleus of *C. roseus *cells was obtained using a bZIP63-YFP^N ^and bZIP63-YFP^C ^double transformation [38] (Figure 8a) whereas the absence of non specific interaction of CrSGD with bZIP63 was checked with the combinations of double transformation of constructs from both proteins fused either in N-terminus or C-terminus of YFP^N ^and YFP^C^, respectively (Figure 8b,c,d,g). The combinations of double transformation of the four CrSGD BiFC constructs always showed a specific nuclear fluorescence signal suggesting that CrSGD was able to at least dimerise irrespective to the accessibility of its extremities (Figure 8e,f,h,i). However, it was absolutely necessary that both coexpressed fusion proteins had the CrSGD C-terminus extremity accessible (YFP^N^-CrSGD and YFP^C^-CrSGD) to display the typical punctuated/fusiform fluorescence pattern of the multimerised CrSGD (Figure 8i). Taken all together, these results demonstrated that CrSGD was addressed to the nucleus using a bipartite NLS that was both necessary and sufficient for the nuclear targeting. They also suggested that CrSGD was able to multimerise in this compartment, as well as in the cytoplasm, when the NLS was deleted, via a sequence located upstream of the C-terminal NLS. This sequence had to remain accessible to promote CrSGD multimerisation since no multimerised organisation could be observed when CrSGD was fused to the N-terminal end of GFP (Figure 6a-h; Additional file 3a-d) or split-YFP (Figure 8e,f,h). Previous work under native conditions described CrSGD as a more than 1,500 kDa multimerised complex [1,37], although its inclusion in large aggregates has not been described before. Plant b-glucosidases have been described to form multimers or to be included in insoluble aggregates in the cytosol and the chloroplast [39-41]. So far few plant b-glucosidases have been observed in the nucleus [42,43] and, to our knowledge, none were found in aggregates. The observed CrSTR and CrSGD subcellular compartmentation is in agreement with the potential for the strictosidine pool to be activated following strictosidine-SGD reunion during herbivore feeding.

### The conserved differential localisation of STR and SGD from *R. serpentina *and *C. roseus *suggests a common strictosidine activation mechanism in Apocynaceae

**Figure 9:**
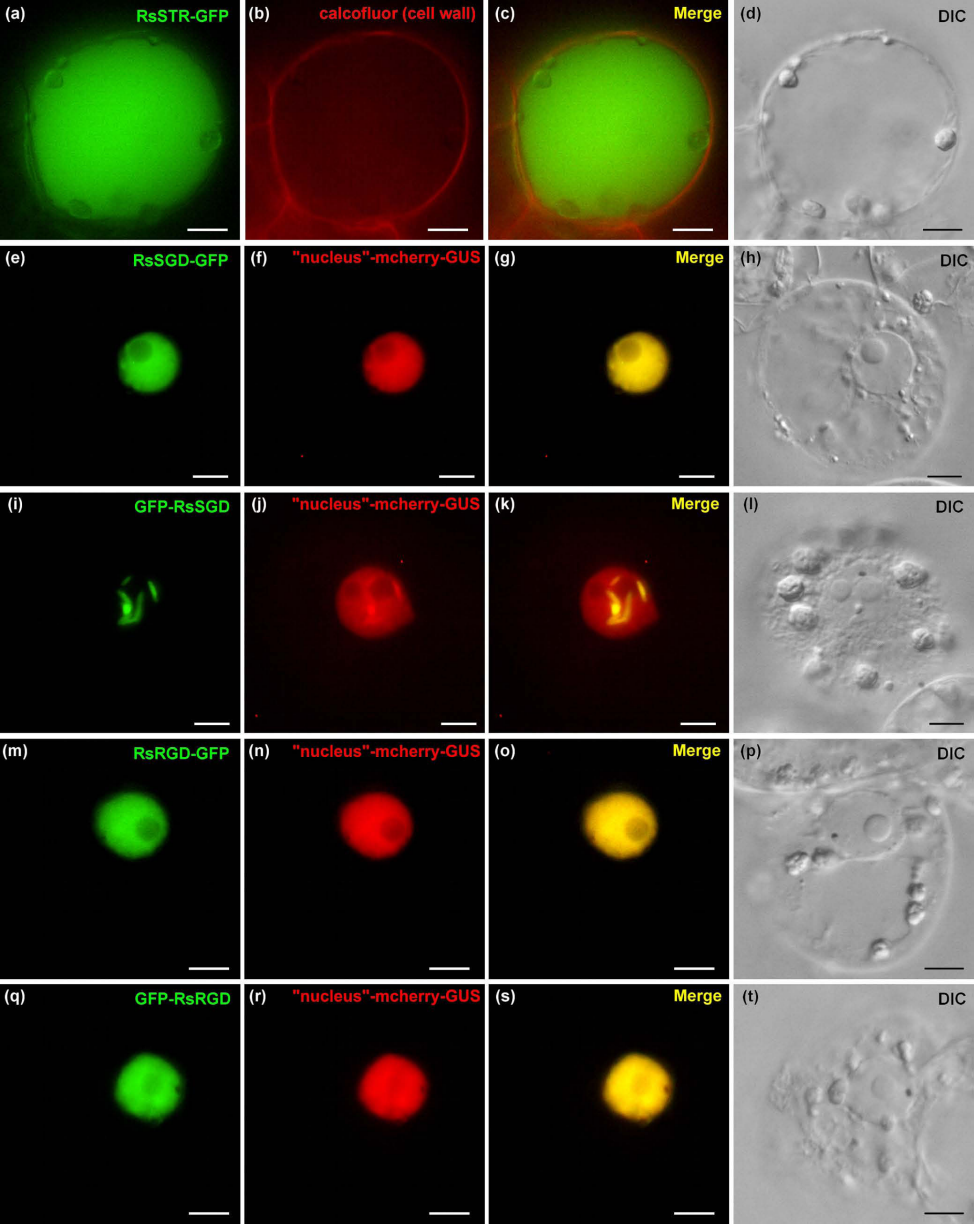
**The subcellular localisation of STR and SGD isolated from *R. serpentina *is conserved but the RsRGD does not aggregate in the nucleus**. Undifferentiated *C. roseus *cells were co-transformed to express RsSTR, RsSGD and RsRGD GFP fusions (1^st ^column) and a nucleus marker (2^nd ^column). The merged image and the DIC morphology are presented in the 3^rd ^and 4^th ^columns, respectively. Note that both RsSTR and RsSGD displayed exactly the same fluorescence patterns as CrSTR and CrSGD, respectively. Note also that RsRGD-GFP and GFP-RsRGD displayed a diffuse nuclear fluorescence pattern reinforcing the specificity of the aggregation of CrSGD and RsSGD. Bar: 10 μm.

With the sequences of STR and SGD from *R. serpentina *also in hands (Figure 1), we studied the subcellular localisation of these enzymes in undifferentiated *C. roseus *cells. RsSTR was targeted to the vacuole in the same way as CrSTR (Figure 9a-d) in agreement with the predicted N-terminal signal peptide (1-MAKLSDSQTMALFTVFLLFLSSSLALS-27) and vacuolar sorting-like peptide (28-SPIL-31). Moreover, RsSGD was localised to the nucleus with the same fluorescence pattern as CrSGD according to the orientation of the fusion (Figure 9e-l) in agreement with the predicted C-terminal bipartite NLS (514-A**KR**RREEAQVELV**KR**Q**K**T-532). This common segregation of vacuolar STR and nuclear SGD suggested that the proposed strictosidine activation-mediated defence process could be a common characteristic of other strictosidine-accumulating Apocynaceae. The discrepancies between the observed common localisation of CrSGD and RsSGD as compared to the previous hypothesis of the CrSGD ER localisation could be either due to the low resolution of the strictosidine-induced yellow fluorescence pattern that made it difficult to distinguish the nucleus from the ER [1], or to the fact that this yellow fluorescence could also correspond to the product of an unidentified ER-located MIA biosynthetic enzyme positioned further downstream in the MIA pathway. All together, CrSGD and RsSGD are now considered to accumulate as multimerised aggregates within the nucleus. It may first appear unusual to find metabolic enzymes within the nucleus. However, some examples have already been described. The flavonoid biosynthetic enzymes chalcone synthase (CHS) and chalcone isomerase (CHI) in *Arabidopsis thaliana *[44], and most interestingly, plant b-glucosidases in *Olea europaea *and *Medicago truncatula *[42,43] have all previously been reported to be localised within the nucleus. Finally, in *C. roseus *and *R. serpentina *this nuclear localisation could insure an efficient physical separation of SGD from the vacuolar pool of strictosidine in intact cells even though the nature of the sequestrating organelle (nucleus) appears rather uncommon. In the mean time this dual subcellular compartmentation renders spatially feasible the massive activation of the strictosidine pool upon organelle reunion occurring during herbivore feeding.

### The CrSGD multimerisation is a specific and autonomous mechanism that increases the CrSGD proteolysis resistance

One point that remained to be explored was the significance of the SGD multimerisation. It has previously been reported that the aggregation pattern of some plant-defence-related β-glucosidases helps to stabilise their activity [14,40,41]. We first studied whether the SGD nuclear aggregation was unique among MIA-related β-glucosidases by analysing the targeting pattern of raucaffricine b-D-glucosidase (RsRGD), another *R. serpentina *MIA-related β-glucosidase (Figure 1). Interestingly, RsRGD was also targeted to the nucleus (Figure 9m-t) in agreement with the predicted C-terminal bipartite NLS (521-V**KR**SIREDDEEQVS**SKR**L**R**K-540) attributing additional intriguing importance of this organelle during the MIA biosynthetic pathway. However, both orientations of the fusion (RsRGD-GFP and GFP-RsRGD) displayed a diffuse nuclear fluorescence pattern suggesting that SGD aggregation may be unique among MIA-related β-glucosidases. Therefore, it would be of interest to study the subcellular localisation and potential aggregation of two new β-glucosidases closely related to RGD (and to lesser extend to SGD) that have been implicated in terpenoid-isoquinoline alkaloid biosynthesis in *Psychotria ipecacuanha *[45] and for which a predicted C-terminal NLS has been identified (data not shown).

**Figure 10:**
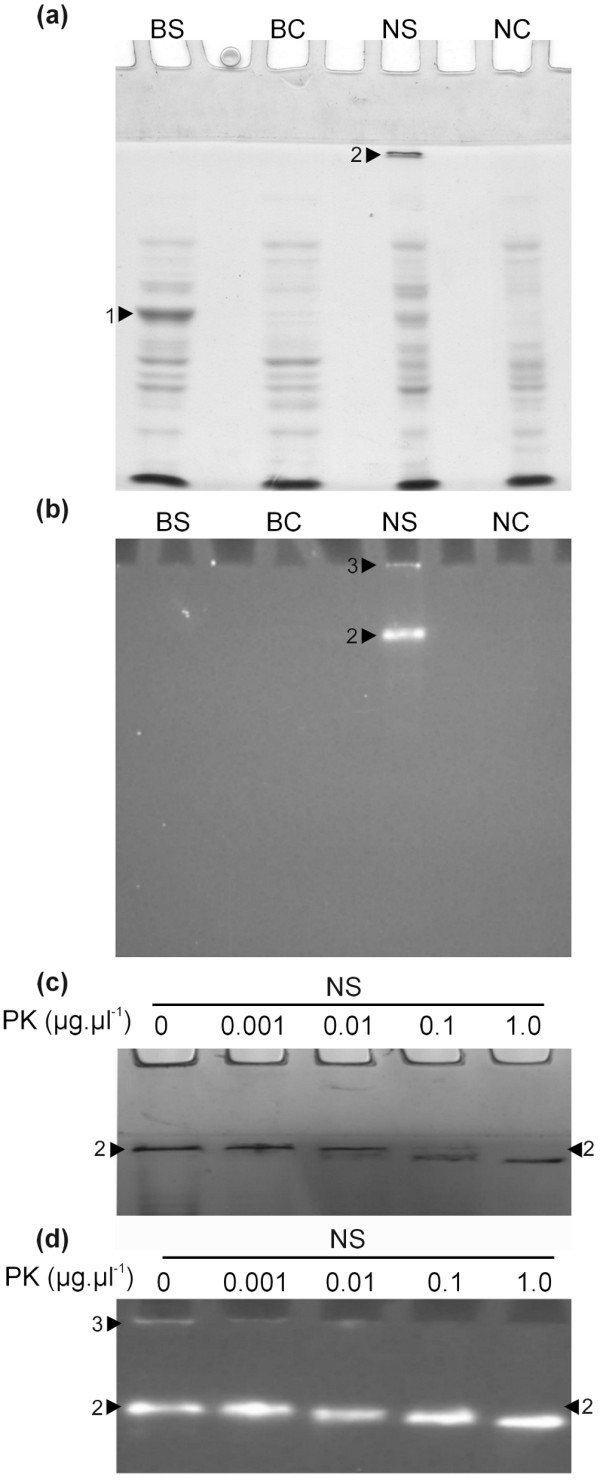
**The native recombinant CrSGD is an autonomously formed supramolecular multimerised complex with a highly stable β-glucosidase activity**. CrSGD was expressed as a recombinant protein in *E. coli *and total *E. coli *protein extracts were analysed by SDS-PAGE (a) or native PAGE (b-d) followed by Coomassie blue staining (a,c) or by β-glucosidase zymogram (b,d) without (a-b) or with (c-d) proteinase K (PK) treatments. The *E. coli *protein extracts from cells transformed with CrSGD-over expressing vector (S) or from control cells transformed with an empty vector (C) were boiled before electrophoresis (B) or native (N). Band annotations: 1, denatured CrSGD present as a monomer; 2, multimerised CrSGD that hardly entered the resolving gel; 3, supramolecular CrSGD that barely enter the stacking gel. Note that the multimerised and supramolecular CrSGD forms (2 and 3) present a specific β-glucosidase activity that is highly stable following PK treatments.

Previous reports have identified specific proteinaceous β-glucosidase aggregating factors that mediate the formation of β-glucosidase large insoluble aggregates in several systems [14,39,46,47]. In order to determine whether SGD aggregation could be under the control of one or more specific aggregating factors in *C. roseus*, we studied the localisation of CrSGD in onion epidermis, a remote non MIA-producing plant model. Interestingly, CrSGD was targeted to the nucleus (Additional file 3i-l) with a specific aggregation pattern when its C-terminus extremity was accessible in the fusion protein (Additional file 3m-p) suggesting that an autonomous aggregation could occur. In addition, we also evaluated the ability of recombinant CrSGD produced in *E. coli *to multimerise *in vitro*. Therefore, we performed EMSA experiments (Figure 10a) with corresponding zymograms using 4-methylumbelliferyl-β-d-glucoside (MUG) as a fluorogenic b-glucosidase generic substrate (Figure 10b). While comparing boiled protein extracts from *E. coli *transformed with either the CrSGD-overexpressing vector (lane BS) or the empty vector negative control (lane BC), CrSGD appeared as a major monomer band (1) (Figure 10a). In unboiled native samples, it was mostly converted into a low mobility band (2) which barely entered the gel. This band represents a native multimerised form of CrSGD since it was specific to the CrSGD-overexpressing vector condition (Figure 10a, lanes NS and NC), pointing once again to autonomous SGD aggregation. The β-glucosidase zymogram (Figure 10b) revealed that the multimerised CrSGD (2) constituted an active enzymatic complex as did a supramolecular form of CrSGD (3) that was undetectable with Coomassie blue staining (Figure 10a-b). The β-glucosidases aggregates are often active [47] but the physiological role of this aggregation remains unclear. Previous authors have proposed that this aggregation provides a protective role against proteases [48] and against herbivore chemical diversion strategies [49]. By incubating recombinant CrSGD enriched protein extracts with increasing amount of proteinase K (PK), we clearly demonstrated the low susceptibility of multimerised CrSGD to protein hydrolysis attack, since PK treatment only caused a slight decrease of the multimer size without affecting its apparent activity (Figure 10c-d). The autonomous SGD multimerisation may thus protect this enzyme from herbivore proteases. Indeed, the saliva of the herbivorous butterfly *Heliconius melpomene *has been shown to be enriched with proteases with high proteolytic activity [50]. Furthermore, proteases have been identified in the midgut secretions of various herbivorous insect species.

## Conclusions

### Physiological and ecophysiological implications

**Figure 11:**
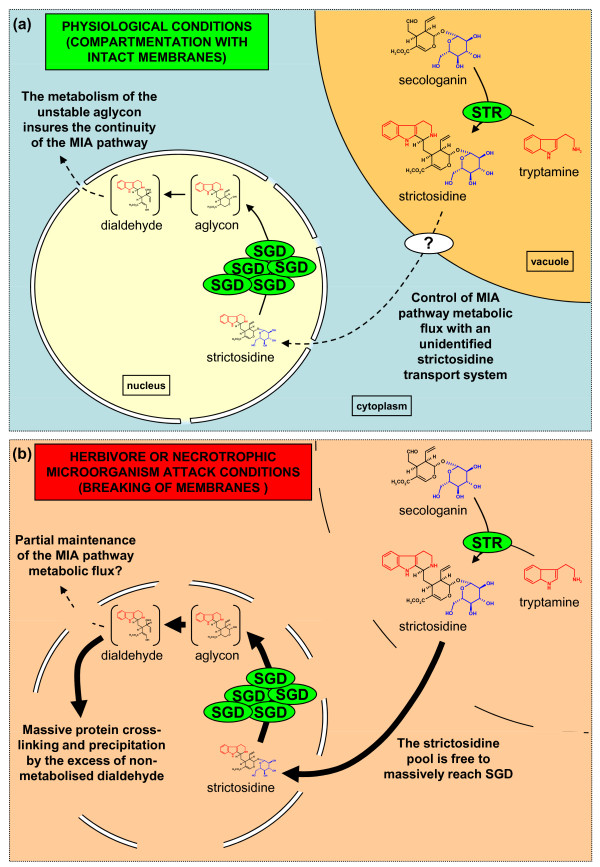
**Working model showing the physiological relevance and the potential plant-defence implications of the vacuole-to-nucleus strictosidine activation**. (a) The subcellular compartmentation of STR and SGD is displayed illustrating that an unknown transportation system of strictosidine across the tonoplast (labelled with "?") constitutes a potential important rate limiting step for the flux of MIA biosynthetic pathway. In *C. roseus*, this part of the pathway is specifically localised to the epidermis of aerial organs. According to the drastic consequences of the CrSGD-mediated massive strictosidine activation (demonstrated by the *in vitro *experience), this potential rate limiting step appears as a mean for the cells to control the rate of formation of toxic dialdehyde following the deglucosylation of strictosidine by SGD in the nucleus in relation with the metabolic capacity of the next MIA biosynthetic enzymes. (b) In the case of herbivore feeding or necrotrophic pathogen attack, this subcellular compartmentation may be mechanically or enzymatically disrupted. As a consequence, the massive deglucosylation of the strictosidine pool by highly stable SGD aggregates leads to the overproduction of the dialdehyde form to such a level that the MIA biosynthetic machinery is not fully able to take in charge. Massive protein cross-linking and precipitation could therefore be a potential mean for the plant to deter the herbivores and/or the necrotrophic microorganism from their feeding habit.

The physiological implications of these results were summarised on a working model (Figure 11). Under normal physiological conditions leading to MIA biosynthesis, the sequestration of strictosidine biosynthesis within the vacuole and its subsequent deglucosylation within the nucleus by a stable supramolecular SGD complex implies that an unknown transportation system of strictosidine across the tonoplast plays an important role in the control of the MIA biosynthetic flux (Figure 11a). Members of the ATP binding cassette (ABC) transporter superfamily have been demonstrated to recruit various alkaloids including the MIA heterodimers vinblastine and vincristine and could therefore be interesting candidates for such transportation [51]. This unknown transportation step appears as highly rate-limiting during the MIA biosynthetic pathway since we showed that MeJa and Ethephon treatments led to an important increase of the strictosidine pool whereas the level of vindoline and catharanthine slightly decreased (Figures 1 and 5). At the moment, apart from an efficient physical separation of SGD from the vacuole-accumulated strictosidine which circumvents the potential deleterious effect of a massive activation of the strictosidine pool, the physiological reason for the intriguing SGD and RGD nuclear sequestration appears unclear. The role of SGD multimerisation may be to prevent both a potential leakage of SGD into the cytoplasm by passive diffusion and as a means to stabilise its enzymatic activity upon potential proteolysis attack. Therefore, under normal physiological conditions, the control of strictosidine vacuolar efflux could insure that the level of activated dialdehyde, produced following strictosidine deglucosylation in the nucleus, is not too high to be fully metabolised by the following steps of the MIA biosynthetic pathway (Figure 11a). At this stage, since the implication of metabolons during plant secondary metabolisms is growingly recognised [52], a tempting hypothesis is that an unknown enzyme could be in close proximity to the site of dialdehyde production in order to rapidly metabolise this activated metabolite, possibly constituting a nuclear metabolon with SGD. In this respect it is interesting to note that pioneer studies of the MIA ajmalicine biosynthetic pathway referred to a so called "ajmalicine synthetase" large multienzyme complex that could encompass the glucosidase activity [53].

In *C. roseus *young leaves, a strictosidine pool constitutively exists in the mM range and is 10-fold increased following hormonal treatment mimicking herbivore and/or necrotrophic microorganism attack whereas slight decrease is observed for vindoline and catharanthine, positioned downstream along the MIA pathway (Figures 1 and 5). This is in agreement with a potential important triggering role for the strictosidine vacuolar pool during herbivore and/or pathogen attack. The second part of this model constitutes a so-called "nuclear time bomb" (Figure 11b). We propose that the massive activation of the strictosidine vacuolar pool by the nuclear SGD complex could occur following cellular disruption, for instance during herbivore feeding or necrotrophic pathogen attack, and that the induced protein cross-linking and precipitation could be a mean for the plant to deter some herbivores from their feeding habit in a similar manner to the *Ligustrum*/oleuropein system[19]. The exact role of protein cross-linking and precipitation in the defence mechanism is here not fully understood partially due to the lack of knowledge on Apocynaceae aggressors. However, induced protein cross-linking and precipitation could be either deleterious for the herbivore/necrotrophic pathogen enzymatic activities and/or could lead to decrease of nutritive value of the food as previously reported for the *Ligustrum*/oleuropein system [19]. In the latter case, the larva of *Brahmaea wallichii*, a *Ligustrum *specialist herbivore has been shown to avoid the plant defence strategy by an adaptive evolution. In this instance, very high concentration of free glycine are found in the larval digestive juice which quench the protein-cross linking effect of the activated oleuropein aglycon [54]. To our knowledge, no such specialist herbivore has been described in *C. roseus *and *R. serpentina *[55]. Such an activation of strictosidine is probably only a part of the plant defence strategies developed in these species given their metabolomic high complexity including numerous directly toxic compounds [7-9,55].

## Methods

### Cloning procedures

*CrSTR *(GeneBank accession CAA43936), *CrSGD *(AAF28800), *RsSTR *(CAA44208), *RsSGD *(CAC83098) and *RsRGD *(AAF03675) cDNAs were amplified using *Pfu *high fidelity DNA polymerase (Promega) from reverse-transcribed total RNA and were fully sequenced. All primers and plasmids used for cloning are listed in Additional file 5.

### Recombinant CrSGD production

The *CrSGD *full-length open reading frame was amplified with the pQE-SGD-Bam and pQE-SGD-Hind oligonucleotides and further cloned in pQE-30 (Qiagen) (Additional file 6). The recombinant protein was expressed in *E. coli *JM109 strain during 4 h at 30°C following addition of 1 mM isopropyl-*β*-D-galactoside during the exponential growth step of the bacterial cell culture. The SGD-expressing and the control (empty vector) *E. coli *cells were collected by centrifugation (15 min, 5,000×g) and resuspended in activity buffer [100 mM sodium phosphate buffer pH 6.55, PMSF (1 mM), EDTA-free protease inhibitor cocktail at working concentration (Roche, Meylan, France)]. The recombinant CrSGD-enriched soluble and the control protein fractions were recovered following a sonication-mediated *E. coli *disruption and centrifugation (15 min, 25,000×g).

### *In vitro *assays of strictosidine activation and BSA cross-linking

All the assays (60 μl) were conducted under continuous shaking during 2 h at 30°C. The reaction mixture consisted in activity buffer and 0.05% (w/v) BSA (Fraction V, Sigma A7906) to which was either added 10 mM strictosidine (Phytoconsult, Leiden, The Netherlands); 10 mM strictosidine and 18.5 μg of protein extract from *E. coli *control cells; 10 mM strictosidine and 18.5 μg of protein extract from SGD-expressing *E. coli *cells; 10 mM tryptamine (Sigma); 10 mM secologanin (Phytoconsult, Leiden, The Netherlands); 10 mM glutaraldehyde (Euromedex, Mundolsheim, France). The tubes were photographed and centrifuged during 1 min at 10,000×g. Ten μl of the supernatants were analysed by SDS-PAGE (3% stacking gel, 10% resolving gel) and Coomassie blue staining to monitor BSA cross-linking and precipitation. In the three strictosidine-containing conditions, 1 μl of the supernatant was mixed in 388 μl of methanol for subsequent HPLC monitoring of strictosidine using a previously described system [56] with the following modifications. The solvent system was aqueous phosphoric acid (0.1% w/v; eluent A) and acetonitrile (eluent B), running as a gradient from 10% B to 27% B within 15 min, to 40% B within 45 min: the flow-rate was 0.5 ml/min and detection at 220 nm. Strictosidine was identified and quantified according to its UV spectrum and retention time by comparison with authentic standard (Phytoconsult, NL).

### Monitoring of strictosidine, vindoline and catharanthine contents in young leaves following hormonal treatments

Periwinkle seeds (*C. roseus *cv. 'Pacifica pink') were surface sterilized by incubation in ethanol (70% v/v) for 2 min, in NaOCl (2.5%) for 20 min at 200 rpm and then washed three times in sterile bi-distilled water. Seeds were subsequently incubated for 24 h in sterile water under darkness at 25 ± 2°C, transferred on static Murashige & Skoog (MS) culture medium for 3 d under darkness at 25 ± 2°C and then exposed to cool white fluorescent light (45 μmol m^-2 ^s^-1^) with a 16 h/8 h photoperiod at 25 ± 2°C. Six-week-old seedlings were transferred singly to sterile magenta box with 50 ml of solid MS culture medium diluted 1:10. After 13 weeks of culture, the plants were transferred to six well plates containing in each well 5 ml of liquid MS culture medium diluted 1:10. The hydroponic plants were treated with methyljasmonate (200 μM) and ethephon (100 μM) at the root level. Each treatment was tested in two independent experiments, each consisting of three replicates. Lyophilized leaves were ground with pillar and mortar and 10 mg were extracted in 400 μl methanol. Plant material and solvent were shaken at 1200 rpm for 1 h and centrifuged at 18,000×g for 5 min. The supernatant was used for subsequent HPLC analyses as described above. The three analysed MIA were identified and quantified according to their UV spectra and retention times by comparison with authentic standards (Strictosidine, Phytoconsult, NL; vindoline and catharanthine, Gedeon Richter ltd., Budapest, HU).

### *In situ *hybridisation

A pBluescript II SK+ vector containing the 1.9 Kb CrSGD full length cDNA (pBS-CrSGD) [1] was used for the *in vitro *transcription of digoxigenin-labelled CrSGD riboprobes. Transcription of the antisense CrSGD riboprobe was realised with the T7 RNA polymerase (Promega) and a *Bam*HI linearised plasmid as a template using digoxigenin-UTP according to the manufacturer's instruction (Roche, Meylan, France) whereas the sense CrSGD riboprobe was similarly transcribed using the T3 RNA polymerase (Promega) and a *Xho*I-linearised pBS-CrSGD. CrSTR and CrD4H riboprobes were described previously [28]. The hydrolysis of riboprobes, the whole *in situ *hybridisation protocol and the microscope analysis were realised as previously described [21,25,27].

### GFP and YFP constructs for CrSTR and CrSGD localisation studies

Plasmids expressing GFP and/or YFP fusion proteins of CrSTR and CrSGD including their orientation, truncated or mutated variants were constructed using the plasmids and the procedures previously described [24]. Details on primers and on cloning procedures are listed in Additional file 5.

### YFP constructs for BiFC studies of CrSGD interactions

For BiFC assays, the CrSGD coding sequence was amplified with primers SGD-GFP-C-S and SGD-GFP-C-AS (Additional file 5) and cloned via *Spe*I in frame with the 5' or 3' ends of the coding sequence of the N-terminal (YFP^N^, amino acids 1-173) and C-terminal (YFP^C^, amino acids 156-239) fragments of YFP. The pSPYNE(R)173 and pSPYCE(MR) plasmids [57] were used to generate constructs for YFP^N^-SGD and YFP^C^-SGD expression, respectively. The split-YFP coding sequence and Nos terminator of the pSPYNE173 and pSPYCE(M) plasmids [57] were removed by a *Spe*I/*Eco*RI digestion and cloned into the pSCA-cassette YFPi plasmid [24] pre-digested by *Spe*I/*Eco*RI to remove the full length coding sequence of YFP and the terminator. The resulting pSCA-SPYNE173 and pSCA-SPYCE(M) plasmids were used for the expression of SGD-YFP^N ^and SGD-YFP^C ^fusion proteins, respectively.

### GFP constructs for RsSTR, RsSGD and RsRGD localisation studies

For studying the subcellular localisation of RsSTR, RsSGD and RsRGD, the coding sequence of the three enzymes were cloned in the pSCA-cassette-GFPi plasmid to express a GFP-fused protein (Additional file 7).

### Organelle markers

A set of organelle markers was used for co-transformation studies with the STR and SGD constructs. "ER"-mcherry (CD3-960), "ER"-YFP (CD3-958) and "plastid"-CFP (CD3-994) markers [58] were obtained from the ABRC http://www.arabidopsis.org. The YFP-GUS cytoplasmic marker was previously described [24]. The CFP-GUS cytoplasmic marker and the CFP nucleocytoplasmic marker were generated following amplification of the CFP coding sequence using primers CFP-for and CFP-rev (Additional file 8). The YFP coding sequence of the pSCA-cassette YFPi and pSCA-cassette YFP-GUS plasmids [24] was then substituted by the CFP coding sequence *via *a *Bgl*II/*Nhe*I cloning step to create the cytoplasmic and nucleocytoplasmic markers, respectively. Using the same procedure, a pSCA-cassette mcherry-GUS was also created after amplification of the mcherry coding sequence with primers mcherry-for and mcherry-rev (Additional file 8). To construct the "nucleus"-mcherry-GUS and "nucleus"-CFP-GUS markers, the sequence of the nucleoplasmin bipartite NLS [36] has been added at the N-terminal end of both mcherry-GUS and CFP-GUS fusion proteins following annealing of the NLS-nucleo-for and NLS-nucleo-rev primers (Additional file 8) performed as previously described [22], and cloning of the resulting product into *Bgl*II/*Spe*I-linearised pSCA-cassette-mcherry-GUS and pSCA-cassette-CFP-GUS plasmids. Cell wall (cellulose) staining was performed with calcofluor (0.1 mg/ml). For BiFC assays, the bZIP-YFP^N ^and bZIP-YFP^C ^expressing plasmids [38] were obtained from Jörg Kudla http://www.uni-muenster.de/Biologie.Botanik/agkudla/Plasmids.html.

### Biolistic-mediated transient transformation of *C. roseus *cells, *C. roseus *leaves and onion epidermis

GFP fusion constructs were either co-transformed with mcherry or YFP organelle markers and cells transformed with single GFP fusion constructs were further stained with Calcofluor. YFP fusion constructs were co-transformed with CFP organelle markers. BiFC constructs were additionally co-transformed with the "plastid"-CFP marker used as a transformation control. The whole detailed protocol of *C. roseus *cell cultures (co)transformation has been previously optimised and extensively detailed [24]. A similar procedure has been applied for transient transformation of *C. roseus *young leaves (5-10 mm) that were platted onto solid Gamborg B5 medium one hour prior to particle bombardment with abaxial epidermis face-up. For the transient transformation of onion cells, internal epidermis of fresh onion were peeled and placed on solid vitamin-free MS medium and bombarded following the protocol of *C. roseus *cell transformation.

### Brefeldin A treatments

For studying the vacuolar route of CrSTR, the ER-to-cis golgi anterograde endomembrane transport has been inhibited by transferring *C. roseus *platted cells onto a solid Gamborg B5 medium containing 40 μM brefeldin A (Invitrogen) one hour before bombardment. The observations were performed 24 h post-transformation.

### Epifluorescence microscopy

An Olympus BX51 epifluorescence microscope equipped with the Olympus DP50 digital camera and the Cell* imaging software (Soft Imaging System, Olympus) was used for image capture and for merging false-coloured images of transiently (co)transformed XFP expressing cells. Details on the combinations of filter sets used for each application are given in Additional file 9. The morphology of the transformed cells was observed with differential interference contrast (DIC).

### Anti-GFP western blotting

The fusion protein content of CrSGD-GFP and GFP-CrSGD stably transformed *C. roseus *calli, obtained following the previously described protocol [59], was analyzed by immunoblot detection with anti-GFP polyclonal antibodies (Molecular Probes) used at 1/1000 dilution.

### Native PAGE and SDS-PAGE-Coomassie blue staining and native PAGE-β-glucosidase zymograms to study CrSGD aggregation and stability

To study the recombinant CrSGD *in vitro *aggregation, 40 μg of protein extract from *E. coli *control cells and SGD-expressing *E. coli *cells were mixed with SDS-loading buffer (50 mM Tris-HCl, pH 6.8, 0.5% (v/v) β-mercaptoethanol, 5% (w/v) SDS, 10% (v/v) glycerol, 0.01% (w/v) bromophenol blue) and boiled when specified before analysis on 8% SDS-PAGE with subsequent Coomassie blue staining. The β-glucosidase activity of these protein extracts was analysed by subjecting the same amount of protein to 8% native PAGE after addition of native-loading buffer (50 mM Tris-HCl, pH 6.8, 10% (v/v) glycerol, 0.01% (w/v) bromophenol blue). The β-glucosidase zymograms were conducted by equilibrating the gels in 50 mM citrate/100 mM phosphate buffer (pH 5.8) during 10 min with further incubation for 10 min in a developing solution containing 1 mM 4-methylumbelliferyl-β-D-glucoside (MUGlc) as previously described [46]. β-glucosidase activity was visualised under UV irradiation (365 nm). The resistance of CrSGD aggregates and the stability of the β-glucosidase activity towards proteolysis were assayed by incubating 40 μg of protein extract from CrSGD-expressing *E. coli *cells (without EDTA-free protease inhibitor cocktail) with increasing concentrations of proteinase K (Invitrogen) up to 1 μg/μl in a 50 μl reaction mixture. After a one hour-incubation at 37°C, the mixture was subjected to 8% native PAGE either followed by Coomassie blue staining and β-glucosidase zymogram.

## Authors' contributions

GG carried out the GFP/BIFC-imaging studies including all plasmid constructs, the SGD expression, the western blot analysis and EMSA. AL designed and carried out the hormonal treatment assays and HPLC experiments and helped in editing the manuscript. SM participated in the *in situ *hybridisation studies. AG participated in some GFP imaging studies. NB participated in the HPLC experiments. NG participated in the SGD aggregation studies. BSP co-supervised the work and helped in editing the manuscript. VC and VB conceived of the study, carried out its design and coordination, performed part of the experiments and wrote the manuscript. All authors read and approved the final manuscript.

## Authors' Information

Vincent Courdavault is the author to whom material demand should be addressed.

## Supplementary Material

Additional file 1 **CrSTR utilises the secretory pathway to reach the vacuole since the localisation of CrSTR-GFP is sensitive to brefeldin A (BFA) treatment**. Undifferentiated *C. roseus *cells were co-transformed to express CrSTR-GFP and an ER marker as labelled in the 1^st ^and 2^nd ^column, respectively. The merged image and the DIC morphology are presented in the 3^rd ^and 4^th ^columns, respectively. The co-transformed cells were pre-incubated (1^st ^lane) or not (2^nd ^lane) with BFA. Note that following BFA treatment, CrSTR-GFP was retained to the destructurated ER compartment indicating that CrSTR followed an ER-to-golgi-to-vacuole route. Bar: 10 μm. Click here for file

Additional file 2 **Immunoblotting analysis of stably transformed *C. roseus *cell lines expressing CrSGD-GFP or GFP-CrSGD**. (a) An anti-GFP western blot analysis was performed on total protein extracts from SGD-GFP or GFP-SGD stably transformed *C. roseus *cells. The SGD-GFP and GFP-SGD fusion proteins were detected as an approximately 95 kDa polypeptide in close agreement with the predicted size of SGD (63 kDa) and GFP (28 kDa). (b) Epifluorescence profile of the SGD-GFP and GFP-SGD stably transformed *C. roseus *cells. Click here for file

Additional file 3 **The diffuse fluorescence pattern of CrSGD-YFP and the aggregation fluorescence pattern of YFP-CrSGD also occur in the nucleus of *C. roseus *and onion epidermal cells**. *C. roseus *leaves (a-h) and onion epidermis (i-p) were co-transformed to express CrSGD-YFP or YFP-CrSGD (1^st ^column) and organelle markers (2^nd ^column). The merged image and the DIC morphology are presented in the 3^rd ^and 4^th ^columns, respectively. Note that the exact same nuclear fluorescence patterns as those observed in undifferentiated *C. roseus *cells also occur in the CrSGD-specific cells, i.e. the *C. roseus *epidermal cells. Note also that the aggregation pattern of YFP-CrSGD also occurs in MIA non-producing cells, i.e. onion epidermis. Bar: 10 μm. Click here for file

Additional file 4 **Control of the co-transformation efficiency during BiFC experiments displayed in Figure 8**. Undifferentiated *C. roseus *cells were co-transformed with the BiFC constructs labelled on the left (fusions with the YFP^C ^fragment) and on the top (fusions with the YFP^N ^fragment), together with a "plastid"-CFP marker. The co-transformation efficiency of the cells displayed in Figure 8 was evaluated with the "plastid"-CFP marker. This figure displays the CFP channel in the exact same focal plan for the cells analysed by BiFC in Figure 8. bZIP63, positive control; YFP^N^, (aa 1-173); YFP^C^, (aa 156-239). Bar: 10 μm. Click here for file

Additional file 5 **Detail of primer sequences and cloning procedure to generate CrSTR and CrSGD fusions proteins**. Click here for file

Additional file 6 **Primer sequences and cloning procedure to generate the construct for expression of recombinant CrSGD in *E. coli***. Click here for file

Additional file 7 **Detail of primer sequences and cloning procedure to generate RsSTR, RsSGD and CrRGD fusions proteins with GFP**. Click here for file

Additional file 8 **Sequence of primers used to generate organelle markers**. Click here for file

Additional file 9 **Details on the combination of filter sets used for each application**. BP, Band Pass; LP, Long Pass. Click here for file
