# Supplementary material for: Strictosidine activation in Apocynaceae: towards a "nuclear time bomb"?
Source: BMC Plant Biol. 2010 Aug 19;10:182. doi: 10.1186/1471-2229-10-182 (PMC3095312; doi:10.1186/1471-2229-10-182)
Supplement: Additional file 1 — CrSTR utilises the secretory pathway to reach the vacuole since the localisation of CrSTR-GFP is sensitive to brefeldin A (BFA) treatment. Undifferentiated C. roseus cells were co-transformed to express CrSTR-GFP and an ER marker as labelled in the 1st and 2nd column, respectively. The merged image and the DIC morphology are presented in the 3rd and 4th columns, respectively. The co-transformed cells were pre-incubated (1st lane) or not (2nd lane) with BFA. Note that following BFA treatment, CrSTR-GFP was retained to the destructurated ER compartment indicating that CrSTR followed an ER-to-golgi-to-vacuole route. Bar: 10 μm. [file 1471-2229-10-182-S1.PDF]

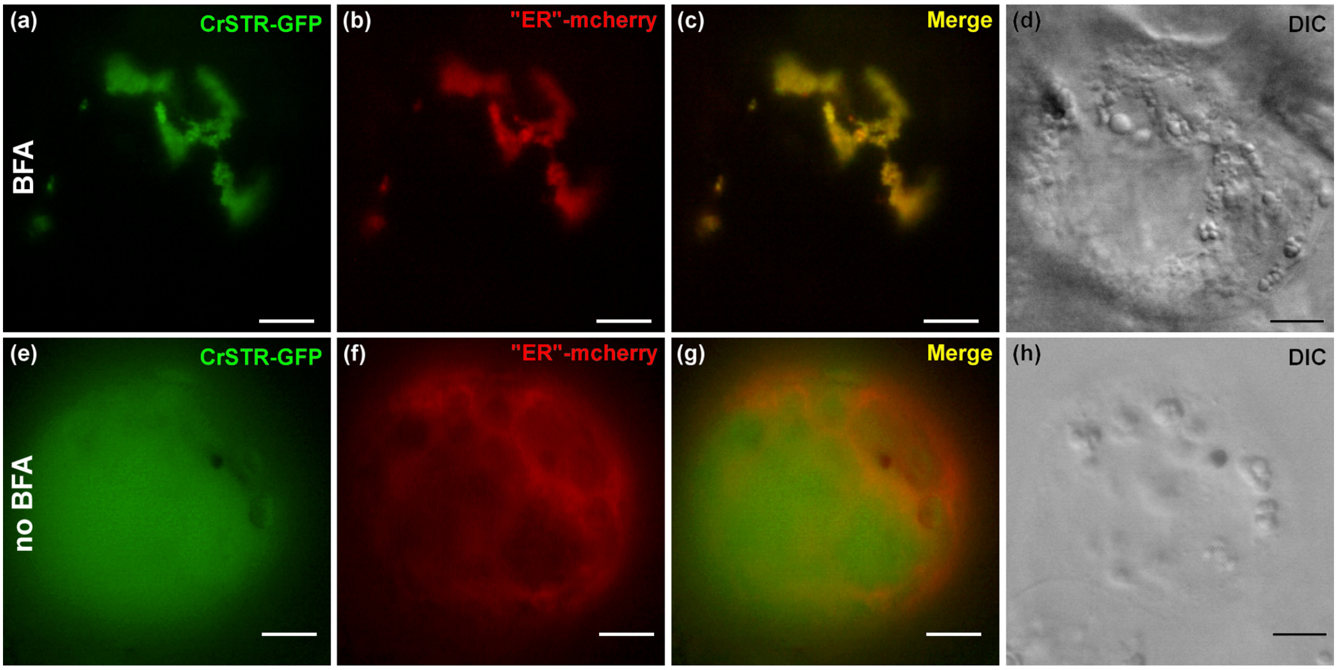

Additional file 1

CrSTR utilises the secretory pathway to reach the vacuole since the localisation of CrSTR-GFP is sensitive to brefeldin A (BFA) treatment.

Undifferentiated *C. roseus* cells were co-transformed to express CrSTR-GFP and an ER marker as labelled in the 1st and 2nd column, respectively. The merged image and the DIC morphology are presented in the 3rd and 4th columns, respectively. The co-transformed cells were pre-incubated (1st lane) or not (2nd lane) with BFA. Note that following BFA treatment, CrSTR-GFP was retained to the destructurated ER compartment indicating that CrSTR followed an ER-to-golgi-to-vacuole route. Bar: 10µm.
