# Supplementary material for: Strictosidine activation in Apocynaceae: towards a "nuclear time bomb"?
Source: BMC Plant Biol. 2010 Aug 19;10:182. doi: 10.1186/1471-2229-10-182 (PMC3095312; doi:10.1186/1471-2229-10-182)
Supplement: Additional file 2 — Immunoblotting analysis of stably transformed C. roseus cell lines expressing CrSGD-GFP or GFP-CrSGD. (a) An anti-GFP western blot analysis was performed on total protein extracts from SGD-GFP or GFP-SGD stably transformed C. roseus cells. The SGD-GFP and GFP-SGD fusion proteins were detected as an approximately 95 kDa polypeptide in close agreement with the predicted size of SGD (63 kDa) and GFP (28 kDa). (b) Epifluorescence profile of the SGD-GFP and GFP-SGD stably transformed C. roseus cells. [file 1471-2229-10-182-S2.PDF]

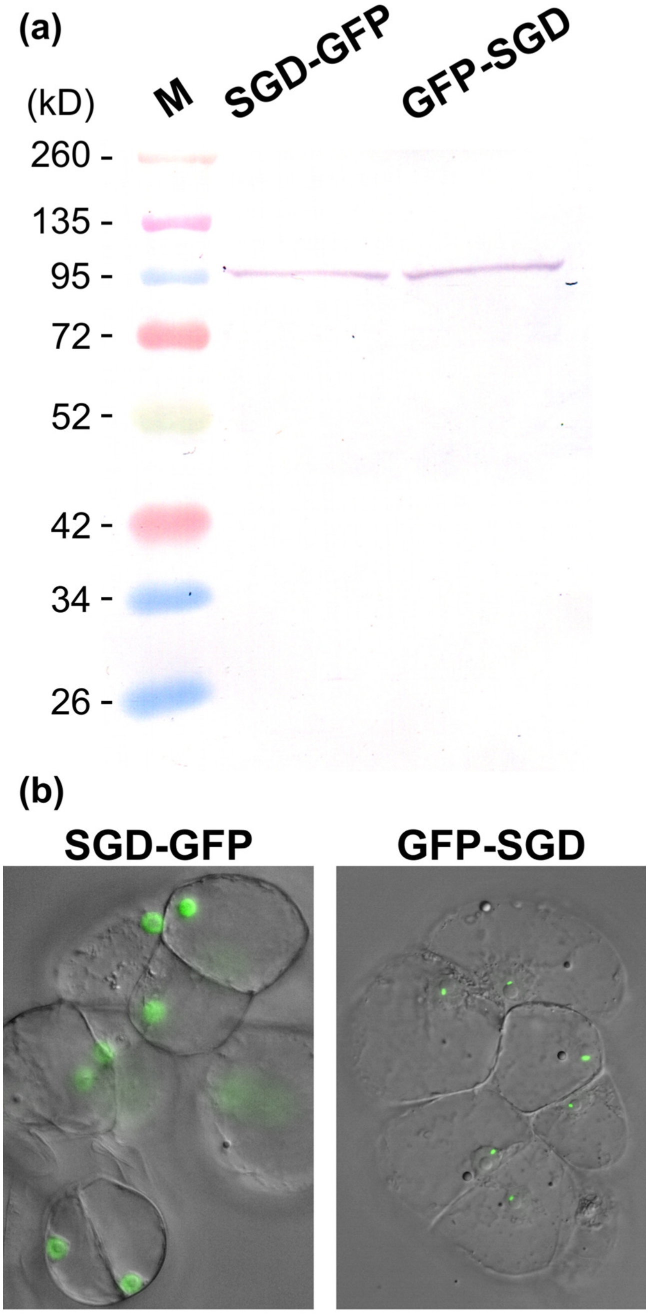

Additional file 2

Immunoblotting analysis of stably transformed *C. roseus* cell lines expressing CrSGD-GFP or GFP-CrSGD.

(a) An anti-GFP western blot analysis was performed on total protein extracts from SGD-GFP or GFP-SGD stably transformed *C. roseus* cells. The SGD-GFP and GFP-SGD fusion proteins were detected as an approximately 95 kDa polypeptide in close agreement with the predicted size of SGD (63 kDa) and GFP (28kDa). (b) Epifluorescence profile of the SGD-GFP and GFP-SGD stably transformed *C. roseus* cells.
