# Supplementary material for: Strictosidine activation in Apocynaceae: towards a "nuclear time bomb"?
Source: BMC Plant Biol. 2010 Aug 19;10:182. doi: 10.1186/1471-2229-10-182 (PMC3095312; doi:10.1186/1471-2229-10-182)
Supplement: Additional file 3 — The diffuse fluorescence pattern of CrSGD-YFP and the aggregation fluorescence pattern of YFP-CrSGD also occur in the nucleus of C. roseus and onion epidermal cells. C. roseus leaves (a-h) and onion epidermis (i-p) were co-transformed to express CrSGD-YFP or YFP-CrSGD (1st column) and organelle markers (2nd column). The merged image and the DIC morphology are presented in the 3rd and 4th columns, respectively. Note that the exact same nuclear fluorescence patterns as those observed in undifferentiated C. roseus cells also occur in the CrSGD-specific cells, i.e. the C. roseus epidermal cells. Note also that the aggregation pattern of YFP-CrSGD also occurs in MIA non-producing cells, i.e. onion epidermis. Bar: 10 μm. [file 1471-2229-10-182-S3.PDF]

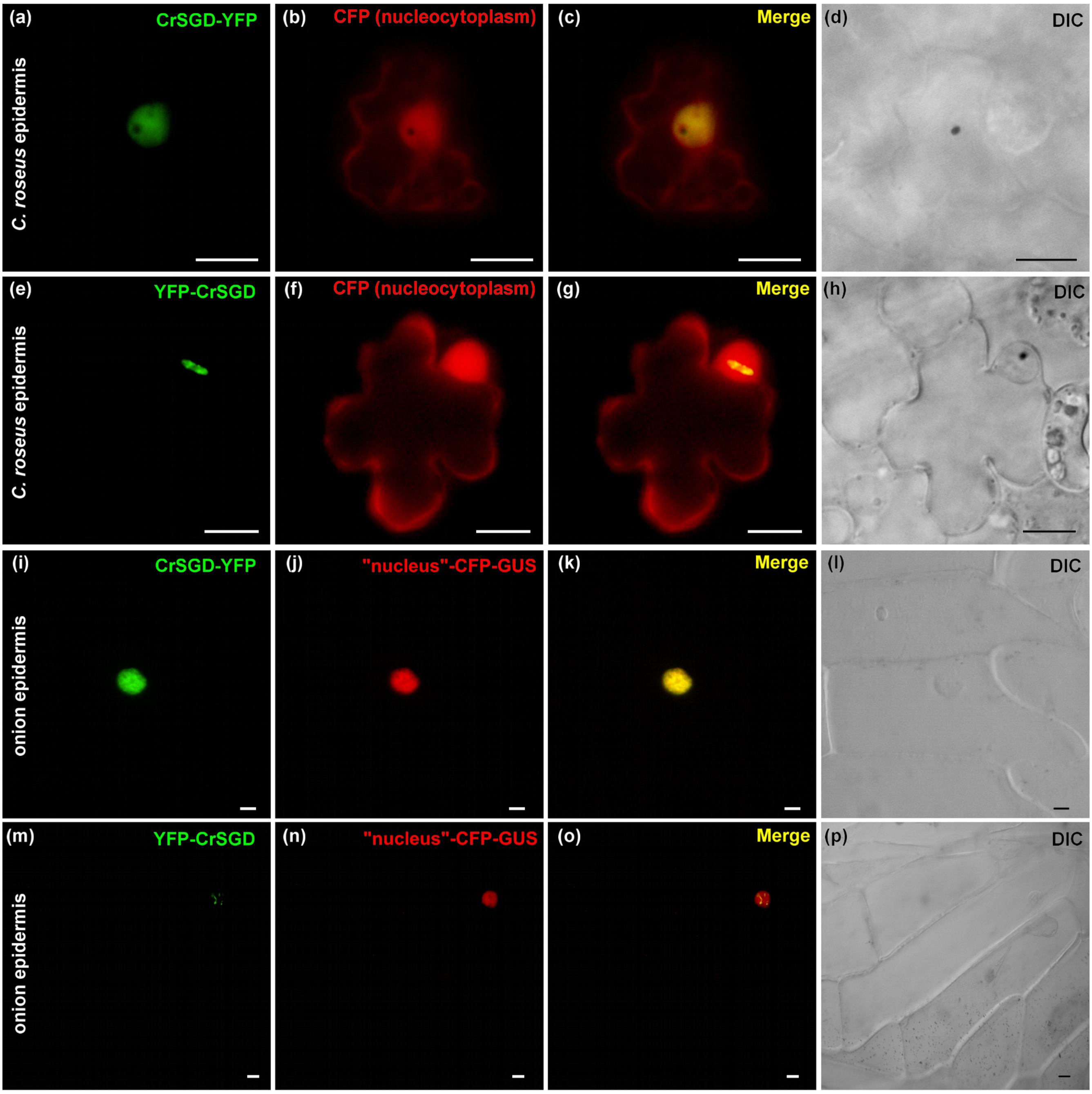

Additional file 3

The diffuse fluorescence pattern of CrSGD-YFP and the aggregation fluorescence pattern of YFP-CrSGD also occur in the nucleus of *C. roseus* and onion epidermal cells.

C. roseus leaves (a-h) and onion epidermis (i-p) were co-transformed to express CrSGD-YFP or YFP-CrSGD (1st column) and organelle markers (2nd column). The merged image and the DIC morphology are presented in the 3rd and 4th columns, respectively. Note that the exact same nuclear fluorescence patterns as those observed in undifferentiated *C. roseus* cells also occur in the CrSGD-specific cells, i.e. the *C. roseus* epidermal cells. Note also that the aggregation pattern of YFP-CrSGD also occurs in MIA non-producing cells, i.e. onion epidermis. Bar: 10µm.
