# Supplementary material for: Strictosidine activation in Apocynaceae: towards a "nuclear time bomb"?
Source: BMC Plant Biol. 2010 Aug 19;10:182. doi: 10.1186/1471-2229-10-182 (PMC3095312; doi:10.1186/1471-2229-10-182)
Supplement: Additional file 4 — Control of the co-transformation efficiency during BiFC experiments displayed in Figure 8. Undifferentiated C. roseus cells were co-transformed with the BiFC constructs labelled on the left (fusions with the YFPC fragment) and on the top (fusions with the YFPN fragment), together with a "plastid"-CFP marker. The co-transformation efficiency of the cells displayed in Figure 8 was evaluated with the "plastid"-CFP marker. This figure displays the CFP channel in the exact same focal plan for the cells analysed by BiFC in Figure 8. bZIP63, positive control; YFPN, (aa 1-173); YFPC, (aa 156-239). Bar: 10 μm. [file 1471-2229-10-182-S4.PDF]

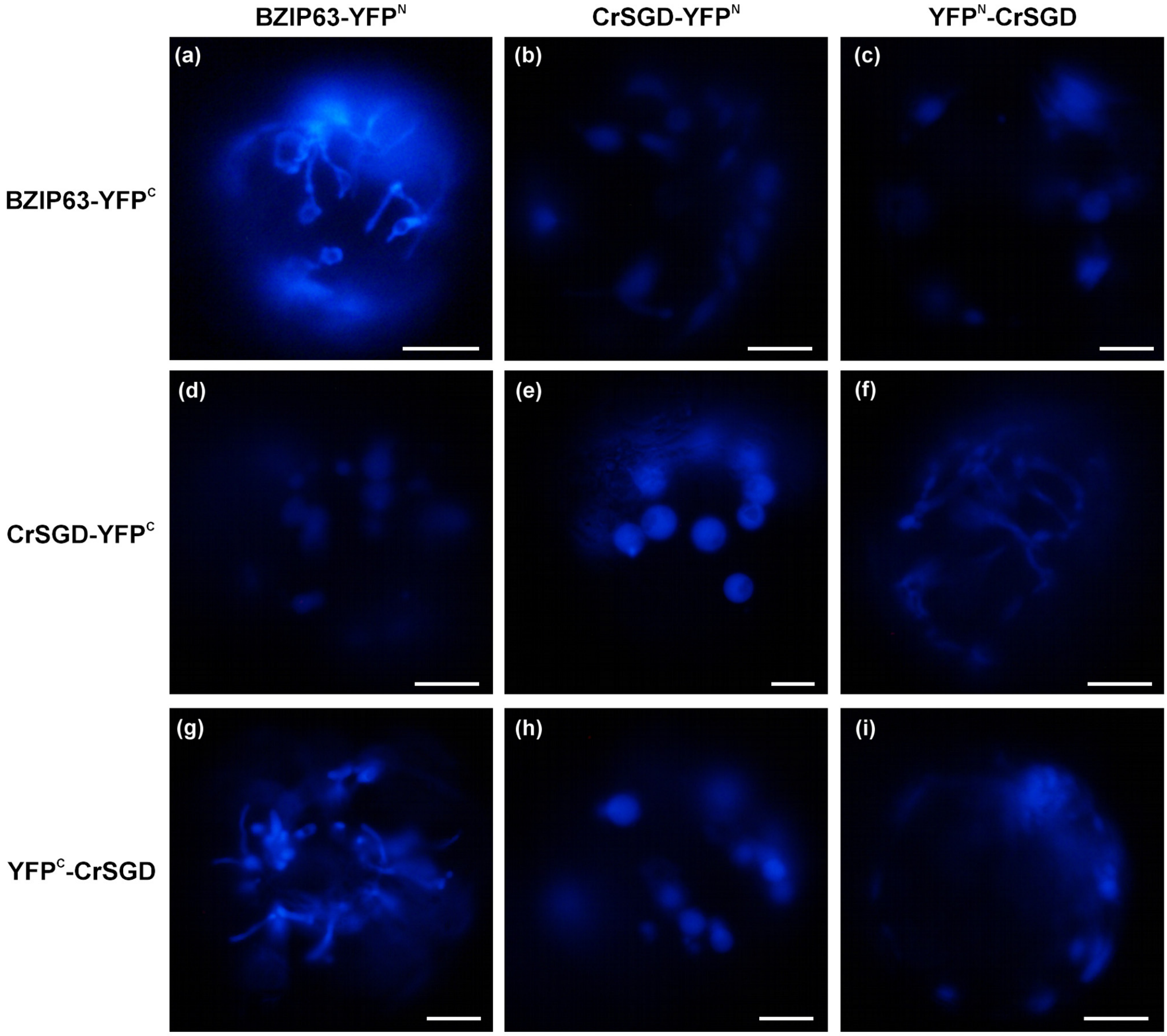

Additional file 4

Control of the co-transformation efficiency during BiFC experiments displayed in Figure 8.

Undifferentiated *C. roseus* cells were co-transformed with the BiFC constructs labelled on the left (fusions with the YFPC fragment) and on the top (fusions with the YFPN fragment), together with a "plastid"-CFP marker. The co-transformation efficiency of the cells displayed in Figure 8 was evaluated with the "plastid"-CFP marker. This figure displays the CFP channel in the exact same focal plan for the cells analysed by BiFC in Figure 8. bZIP63, positive control; YFPN, (aa 1-173); YFPC, (aa 156-239). Bar: 10µm.
