# Supplementary material for: Strictosidine activation in Apocynaceae: towards a "nuclear time bomb"?
Source: BMC Plant Biol. 2010 Aug 19;10:182. doi: 10.1186/1471-2229-10-182 (PMC3095312; doi:10.1186/1471-2229-10-182)
Supplement: Additional file 5 — Detail of primer sequences and cloning procedure to generate CrSTR and CrSGD fusions proteins. [file 1471-2229-10-182-S5.PDF]

<table>
<thead>
<tr><th>Enzyme</th><th>Primer sequence (5’ – 3’)</th><th>Restriction site added</th><th>Plasmids
cloning sites</th><th>Fusion protein</th></tr>
</thead>
<tbody>
<tr><td rowspan="8">CrSTR
(352 AA)</td><td>STR-BglII
GCAGATCTGATGGCAAACTTTTCTGAATCTAAATCC</td><td>BglII</td><td rowspan="2">pSCA-cassette-GFPi
BglII - SpeI</td><td rowspan="2">STR-GFP
(1-352)</td></tr>
<tr><td>STR-SpeI
GCACTAGTGCTAGAAACATAAGAATTTCCCTTGTTA</td><td>SpeI</td></tr>
<tr><td>STR-peptide for
GCAGATCTGATGGCAAACTTTTCTGAATCTA</td><td>BglII</td><td rowspan="2">pSCA-cassette-GFPi
BglII - SpeI</td><td rowspan="2">sp(SPIL)-GFP
(1-41)</td></tr>
<tr><td>STR-peptide-SPIL
GCACTAGTTTCAATAAAAATCTTTTTCAAAATTGG</td><td>SpeI</td></tr>
<tr><td>STR-peptide-SPIL-mut
GCACTAGTTTCAATAAAAATCTTTTTCAACCCTGG
in association with STR-peptide for</td><td>SpeI</td><td>pSCA-cassette-GFPi
BglII - SpeI</td><td>spmut(SPGL)-GFP
(1-41)</td></tr>
<tr><td>STR-del SPIL
GCACTAGTATGAAAAAGATTTTTATTGAAAGCCCTTCC
in association with STR-SpeI</td><td>SpeI</td><td>pSCA-cassette-YFPi
SpeI</td><td>Δsp(SPIL)STR-GFP
(36-352)</td></tr>
</tbody>
<tbody>
<tr><td rowspan="9">CrSGD
(555 AA)</td><td>SGD-GFP-C-S
GGTCTAGAATGGGATCTAAAGATGATCAGTCC</td><td>XbaI</td><td rowspan="2">pSCA-cassette-GFPi
pSCA-cassette-YFPi
SpeI</td><td rowspan="2">SGD-GFP
SGD-YFP
(1-555)</td></tr>
<tr><td>SGD-GFP-C-AS
GCTCTAGAGTATTTTTGCTTCTTGACTAACTCAAC</td><td>XbaI</td></tr>
<tr><td>SGD-GFP-C-AS-stop
GCTCTAGATTAGTATTTTTGCTTCTTGACTAACTC
in association with SGD-GFP-C-S</td><td>XbaI</td><td>pSCA-cassette-GFPi
pSCA-cassette-YFPi
NheI</td><td>GFP-SGD
YFP-SGD
(1-555)</td></tr>
<tr><td>SGD-del-S
GGAGATCTGATGGGATCTAAAGATGATCAGTCC</td><td>BglII</td><td rowspan="2">pSCA-cassette-GFPi
BglII - SpeI</td><td rowspan="2">SGDΔnls
-GFP
(1-536)</td></tr>
<tr><td>SGD-del-AS
GGACTAGTAGCTGTATTCGTAACAAATCCTTC</td><td>SpeI</td></tr>
<tr><td>SGD-GFP-C-S
in association with SGD-del-AS</td><td>XbaI/SpeI</td><td>pSCA-cassette-GFPi
NheI</td><td>GFP-SGDΔnls
(1-536)</td></tr>
<tr><td>SGD-peptide_for
GAGCTAGCAAAAAGAGATTCCGAGAAGAAGATAAACTA</td><td>NheI</td><td rowspan="2">pSCA-cassette-GFPi-GUS
NheI</td><td rowspan="2">GFP-GUS-nls
(537-555)</td></tr>
<tr><td>NLS-SGDrev2
GAGCTAGCGTACCTTAAAGAGCGGTTCAGATCA</td><td>NheI</td></tr>
</tbody>
</table>

Additional file 5: Detail of primer sequences and cloning procedure to generate CrSTR and CrSGD fusions proteins
