# Supplementary material for: Strictosidine activation in Apocynaceae: towards a "nuclear time bomb"?
Source: BMC Plant Biol. 2010 Aug 19;10:182. doi: 10.1186/1471-2229-10-182 (PMC3095312; doi:10.1186/1471-2229-10-182)
Supplement: Additional file 6 — Primer sequences and cloning procedure to generate the construct for expression of recombinant CrSGD in E. coli. [file 1471-2229-10-182-S6.PDF]

Enzyme	Primer sequence (5’ – 3’)	Restriction site added	Plasmids<br>*cloning sites*	Fusion protein
**CrSGD**	pQE-SGD-Bam<br>GCGGATCCATGGGATCTAAAGATGATCAGTCCC	*Bam*HI	pQE-30<br>*Bam*HI - *Hin*dIII	6His-SGD
	pQE-SGD-Hind<br>GCAAGCTTTTAGTATTTTTGCTTCTTGACTAACTCA	*Hin*dIII		

Additional file 6: Primer sequences and cloning procedure to generate the construct for expression of recombinant CrSGD in *E. coli*
