# Supplementary material for: Strictosidine activation in Apocynaceae: towards a "nuclear time bomb"?
Source: BMC Plant Biol. 2010 Aug 19;10:182. doi: 10.1186/1471-2229-10-182 (PMC3095312; doi:10.1186/1471-2229-10-182)
Supplement: Additional file 7 — Detail of primer sequences and cloning procedure to generate RsSTR, RsSGD and CrRGD fusions proteins with GFP. [file 1471-2229-10-182-S7.PDF]

**Enzyme**	**Primer sequence (5’ – 3’)**	**Restriction site added**	**Plasmids** ***cloning sites***	**Fusion protein**
**RsSTR** *(344 AA)*	Rs-STR-GFPfor<br>GTACTAGTATGGCCAAACTTTCTGATTCGCAAACT	*Spe*I	pSCA-cassette-GFPi<br>*Spe*I	RsSTR-GFP
	Rs-STR-GFPrev<br>GTACTAGTATGACTTGAAACAAAAGAATTTCCCTTCTT	*Spe*I		
**RsSGD** *(532 AA)*	Rs-SGD-GFPfor<br>GCACTAGTATGGGCAGCATTGATTCAACAAATGTA	*Spe*I	pSCA-cassette-GFPi<br>*Spe*I	RsSGD-GFP
	Rs-SGD-GFPrev<br>GCACTAGTAGCTTCTTTGAGCAAATCATCGGTTAATT	*Spe*I		
	Rs-SGD-GFPfor<br>*in association with* Rs-SGD-GFPrev	*Spe*I	pSCA-cassette-GFPi<br>*Nhe*I	GFP-RsSGD
**RsRGD** *(540AA)*	Rs-RGD-GFPfor<br>GTACTAGTATGGCAACTCAGAGCAGTGCTGTTATC	*Spe*I	pSCA-cassette-GFPi<br>*Spe*I	RsRGD-GFP
	Rs-SGD-GFPrev<br>GTACTAGTCTTTCTTAATCTCTTGCTTGAAACTTGTTC	*Spe*I		
	Rs-RGD-GFPfor<br>*in association with* Rs-SGD-GFPrev	*Spe*I	pSCA-cassette-GFPi<br>*Nhe*I	GFP-RsRGD

Additional file 7: Detail of primer sequences and cloning procedure to generate RsSTR, RsSGD and CrRGD fusions proteins with GFP
