# Supplementary material for: Strictosidine activation in Apocynaceae: towards a "nuclear time bomb"?
Source: BMC Plant Biol. 2010 Aug 19;10:182. doi: 10.1186/1471-2229-10-182 (PMC3095312; doi:10.1186/1471-2229-10-182)
Supplement: Additional file 8 — Sequence of primers used to generate organelle markers. [file 1471-2229-10-182-S8.PDF]

Name	Primer Sequence (5’-3’)
CFP-for	GAAGATCTGACTAGTATGGTGAGCAAGGGCGAGGAGCTGTTCACC
CFP-rev	GCGCTAGCTCTCTTGTACAGCTCGTCCATGCC
mcherry-for	GAAGATCTGACTAGTATGGTGAGCAAGGGCGAGGAGGA
mcherry-rev	GCGCTAGCGTACAGCTCGTCCATGCCGCCGG
NLS-nucleo-for	GATCTGATGAAGAGACCAGCTGCAACAAAGAAGGCCGGACAAGCTAAAAAGAAAAAGA
NLS-nucleo-rev	CTAGTCTTTTTCTTTTTAGCTTGTCCGGCCTTCTTTGTTGCAGCTGGTCTCTTCATCA

Additional file 8: Sequence of primers used to generate organelle markers
