# Supplementary material for: Strictosidine activation in Apocynaceae: towards a "nuclear time bomb"?
Source: BMC Plant Biol. 2010 Aug 19;10:182. doi: 10.1186/1471-2229-10-182 (PMC3095312; doi:10.1186/1471-2229-10-182)
Supplement: Additional file 9 — Details on the combination of filter sets used for each application. BP, Band Pass; LP, Long Pass. [file 1471-2229-10-182-S9.PDF]

Experiment	Fluorochromes	Filter set name (Reference)	excitation filter (nm)	emission filter (nm)
GFP/calcofluor	GFP	JP1 (Chroma#31039)	460-480	500-520 BP
	calcofluor	UV (Olympus WU2)	330-385	420 LP
GFP/mcherry	GFP	JP1 (Chroma#31039)	460-480	500-520 BP
	mcherry	Texas Red (Olympus U-MWIY2)	545-580	610 LP
GFP/YFP	GFP	sapphire/UV GFP (Chroma#31043)	375-415	490-530 BP
	YFP	JP2 (Chroma#31040)	500-520	540-580 BP
YFP/CFP	YFP	JP2 (Chroma#31040)	500-520	540-580 BP
	CFP	Cyan GFP (Chroma#31044v2	426-446	460-500 BP

Additional file 9: Details on the combination of filter sets used for each application. BP, Band Pass; LP, Long Pass.
